# H_2_O_2_ Induces Major Phosphorylation Changes in Critical Regulators of Signal Transduction, Gene Expression, Metabolism and Developmental Networks in *Aspergillus nidulans*

**DOI:** 10.3390/jof7080624

**Published:** 2021-07-31

**Authors:** Ulises Carrasco-Navarro, Jesús Aguirre

**Affiliations:** Departamento de Biología Celular y del Desarrollo, Instituto de Fisiología Celular, Universidad Nacional Autónoma de México, Apartado Postal 70-242, Ciudad de México 04510, Mexico; ulises.c.n@gmail.com

**Keywords:** ROS signaling, stress, phosphoinositide signaling, MAPK, TOR, nitrogen metabolism

## Abstract

Reactive oxygen species (ROS) regulate several aspects of cell physiology in filamentous fungi including the antioxidant response and development. However, little is known about the signaling pathways involved in these processes. Here, we report *Aspergillus nidulans* global phosphoproteome during mycelial growth and show that under these conditions, H_2_O_2_ induces major changes in protein phosphorylation. Among the 1964 phosphoproteins we identified, H_2_O_2_ induced the phosphorylation of 131 proteins at one or more sites as well as the dephosphorylation of a larger set of proteins. A detailed analysis of these phosphoproteins shows that H_2_O_2_ affected the phosphorylation of critical regulatory nodes of phosphoinositide, MAPK, and TOR signaling as well as the phosphorylation of multiple proteins involved in the regulation of gene expression, primary and secondary metabolism, and development. Our results provide a novel and extensive protein phosphorylation landscape in *A. nidulans*, indicating that H_2_O_2_ induces a shift in general metabolism from anabolic to catabolic, and the activation of multiple stress survival pathways. Our results expand the significance of H_2_O_2_ in eukaryotic cell signaling.

## 1. Introduction

Aerobic organisms generate reactive oxygen species (ROS) during normal metabolism. ROS are O_2_ derived molecules that are produced mainly by partial reduction or excitation. Among these, superoxide is produced during respiration and by dedicated enzymes like NADPH oxidases [1], while cellular H_2_O_2_ is generated by spontaneous or superoxide oxidase-mediated dismutation of superoxide. H_2_O_2_, considered as the most important ROS in redox biology [2], is efficiently eliminated by peroxiredoxins, peroxidases, catalase-peroxidases, and catalases. However, peroxiredoxins are active at <10 μM while catalases are active at much higher H_2_O_2_ concentrations [3]. For many decades, ROS were considered as harmful molecules involved in aging and pathogenic processes [4]. However, we and others have shown that ROS also played regulatory roles in different cellular processes such as bacterial gene regulation [5,6], plant [7] and animal [8] signal transduction, and cell differentiation [9,10].

The importance of ROS in cell differentiation has been documented in several species of filamentous fungi. In *Neurospora crassa*, the differentiation of asexual spores involves three morphogenetic transitions, each preceded by an hyperoxidant state that is characterized by the increased production of ROS [11], the production of a redox imbalance, the oxidation and degradation of total protein as well as the oxidative inactivation of nitrogen assimilation enzymes [9,10,12,13,14,15]. Moreover, specific ROS-producing NADPH oxidases are indispensable for sexual differentiation in *N. crassa* [16,17], *A*. *nidulans* [18], *Podospora anserina* [19], *Sordaria macrospora* [20], and other fungi. In *A. nidulans*, ROS also regulate important cellular processes such as mitochondrial division [21,22] and the antioxidant response [23,24,25,26,27,28].

Many of the direct biological effects of H_2_O_2_ are mediated by the oxidation of critical cysteine or methionine residues in specific proteins [2,29]. Indeed, the inactivation of protein tyrosine phosphatases (PTP) by oxidation of their catalytic cysteine is a well-studied mechanism by which H_2_O_2_ can impact protein phosphorylation [30,31]. In addition, recent evidence indicates that the inactivation of kinases mediated by cysteine oxidation might be a ubiquitous mechanism by which H_2_O_2_ can also regulate protein phosphorylation. This is the case or Aurora kinase A, in which the oxidation of a cysteine (C290) prevents the essential phosphorylation of adjacent T288, in its activation segment. This cysteine, conserved in a group of human CAMK, AGC, and AGC-like kinases, is also important to regulate the activity of fission yeast Srk1 and Pka1 kinases [32].

In *A. nidulans* and other fungi, H_2_O_2_ induces the phosphorylation and nuclear localization of the Hog1/p38 orthologous stress-activated MAPKs SakA and MpkC [23,25], and once in the nucleus, SakA interacts with transcription factor AtfA [33]. Both SakA and AtfA are required for resistance to H_2_O_2_ and both proteins are important for proper catalase *catB* gene induction [25,33]. SakA and AtfA also regulate asexual and sexual development, and mutants lacking either SakA, AtfA, or both proteins show very similar phenotypes. H_2_O_2_ also induces the interaction of SakA and MpkC with several proteins including the SrkA kinase. In response to H_2_O_2_, SrkA is translocated to the nucleus in a SakA-dependent process [22]. In addition to the SakA/MpkC pathway, transcription factors SrrA [28] and NapA [26,27] are necessary for H_2_O_2_ resistance, H_2_O_2_-mediated induction of *catB* and other genes, and for proper development. NapA also regulates the expression of multiple genes during asexual development and regulates carbon utilization [26,27]. The fact that H_2_O_2_ activates the SakA pathway, which is highly conserved and critical for stress survival in eukaryotes, shows the importance of H_2_O_2_ in cell signaling. However, the general knowledge about the pathways involved in ROS perception and ROS signal transduction in filamentous fungi is still very limited.

Phosphorylation is the most ubiquitous posttranslational modification found in proteins [34], and by affecting protein activity, subcellular localization, protein interactions and stability, it impacts virtually all biological processes [35]. Because of this, phosphorylation is a highly regulated process that depends on a balance of kinase and phosphatase activities. Thanks to recent advances in the sensitivity of mass spectrometry analysis, it is possible to perform protein phosphorylation analysis on a global scale [34,35]. In *A. nidulans*, global phosphoproteomic analysis has been used to study MpkA functions in cell wall stress [36], PKA-dependent phosphorylation [37], and general protein phosphorylation [38].

We carried out a global phosphoproteomic analysis to identify the proteins and pathways potentially involved in H_2_O_2_ signaling in *A. nidulans*. At the same time, we present an extensive review of the signal transduction and metabolic pathways that were affected in response to H_2_O_2_. We found that H_2_O_2_ has major effects on the phosphorylation of phosphoinositide, MAPK, TOR, and other kinase signaling networks that control growth, stress responses, and development. Moreover, the effects of H_2_O_2_ in critical metabolic enzymes indicate that a major change in metabolism, from anabolic to catabolic, is induced by H_2_O_2_. Our results provide a set of specific predictions on how ROS affect specific signal transduction pathways that can be experimentally tested.

## 2. Materials and Methods

### 2.1. Strains, Media, and Growth Conditions

*Aspergillus nidulans* strain CLK43 (*pabaA1 yA2 veA1*) was used as the wild type strain to compare with previous results [22]. It was grown at 37 °C in 1% glucose liquid minimal medium with nitrate as the nitrogen source [39]. To determine H_2_O_2_ effects on protein phosphorylation, liquid cultures were treated as reported [22]. Briefly, *A. nidulans* mycelia was grown in liquid culture for 12 h and then treated or not with 10 mM H_2_O_2_ during 10 min. After this, triplicate samples from each condition were frozen with liquid nitrogen and processed for phosphoproteomic analysis.

### 2.2. Protein Extraction

Frozen mycelia was ground to a fine powder in a precooled mortar using liquid nitrogen. The resulting powder was resuspended in 50 mM Tris-HCl buffer pH 7.4 containing 6 M urea, 5 mM DTT, and protease inhibitor (Sigma Aldrich, St. Louis, MO, USA) and phosphatase inhibitor (Thermo Scientific, Rockford, IL, USA) cocktails. Samples were kept on ice in 50 mL conic tubes and homogenized for 5 min with a Tissue Tearor homogenizer (Bioespec Products, INC., Bartlesville, OK, USA). Subsequently, the extracts were transferred to a Branson 2200 sonicator (Branson Ultrasonics, St. Louis, MO, USA) and sonicated for 5 min. The protein extract was clarified by centrifugation at 4000 rpm for 20 min and 4 °C. Soluble proteins were precipitated as reported [40]. Briefly, precipitation was carried out at −20 °C for 6 h, after the addition of 1 volume of 20% trichloroacetic acid in acetone containing 0.14% (*w*/*v*) DTT. The final pellet was washed twice with cold acetone, followed by a final wash with 80% cold acetone. Precipitated proteins were solubilized in 50 mM Tris-HCl buffer pH 8 with 6 M urea. Protein concentration was determined using the Bradford Protein Assay (BioRad, Hercules, CA, USA) and a nano spectrophotometer (Implen, München, Germany).

### 2.3. Protein Digestion and Phosphopeptide Enrichment

An aliquot of 3 mg of protein from each sample was reduced with 10 mM DTT at 37 °C for 1 h. Subsequently, iodoacetamide was added to a final concentration of 15 mM and samples were incubated at room temperature for 30 min in the dark. The samples were diluted with three volumes of 50 mM Tris-HCl buffer pH 8. Trypsin (Sigma Aldrich, St. Louis, MO, USA) was added in a ratio of 1:60 (trypsin: protein) and samples were incubated at 37 °C for 18 h. The resulting peptides were desalted by solid phase extraction with HyperSep SPE columns (Thermo Scientific, Rockwood, TN, USA). Phosphopeptides were enriched using the High-Select™ TiO_2_ Phosphopeptide Enrichment Kit (Thermo Scientific, Rockford, IL, USA), following the manufacturer’s instructions.

### 2.4. Liquid Chromatography Coupled to Tandem Mass Spectrometry (LC-MS/MS) Analysis

LC–MS/MS analysis was carried at the Institut de Recherches Cliniques de Montreal (IRCM), Canada. Prior to LC-MS/MS, protein digests were re-solubilized in 1% acetonitrile/1% formic acid and loaded onto a Self-Pack C18 column installed in the Easy-nLC 1200 system (Proxeon Biosystems, Roskilde, Sjælland, Denmark). The buffers used for chromatography were 0.2% formic acid (buffer A) and 90% acetonitrile/0.2% formic acid (buffer B). Peptides were eluted with a two-slope gradient at a flow rate of 250 nL/min. The LC system was coupled to a Orbitrap Fusion Tribrid™ mass spectrometer (Thermo Scientific, Waltham, MA, USA) through a Nanospray Flex Ion Source. Peptide ions were fragmented in the HCD collision cell and analyzed in the linear ion trap with a target value of 1e4 and a normalized collision energy of 29 V. MS3 scanning was performed upon detection of a neutral loss of phosphoric acid (48.99, 32.66 or 24.5 Th) in MS2 scans. Target ions selected for fragmentation were dynamically excluded for 30 s after two MS2 events.

### 2.5. Database Searching

Protein database searching was performed with Mascot 2.5 (Matrix Science, Boston, MA, USA) against the UniProt *Aspergillus nidulans* protein database (version 4 November 2019). The peak list files were generated with Proteome Discoverer (v2.3) using the following parameters: minimum mass set to 500 Da and maximum mass set to 6000 Da. Mascot was searched with a fragment ion mass tolerance of 0.60 Da and a parent ion tolerance of 10.0 PPM. Cysteine carbamidomethylation was specified in Mascot as a fixed modification. Methionine oxidation and serine, threonine, and tyrosine phosphorylation were specified as variable modifications.

### 2.6. Phosphopeptide and Protein Identification Criteria

Scaffold (version Scaffold_4.11.1, Proteome Software Inc., Portland, OR, USA) was used to validate MS/MS based peptide and protein identifications. The search was performed against a decoy database. Peptide identifications were accepted if they could be established at greater than 90.0% probability by the Peptide Prophet algorithm [41]. Protein identifications were accepted if they could be established at greater than 90.0% probability and contained at least one identified peptide. Protein probabilities were assigned by the Protein Prophet algorithm [42]. For phosphosite identification, a MS3 scanning was performed upon detection of a neutral loss of phosphoric acid (48.99, 32.66 or 24.5 Th) in MS2 scans. Target ions selected for fragmentation were dynamically excluded for 30 s after two MS2 events. For phosphopeptide identification, a false discovery rate (FDR) of 1.0% was established, based on a decoy database.

### 2.7. Bioinformatics Analyses

Functional enrichment analysis was performed using NeVOmics, bioinformatic tool based in Python with R packages [43]. STRING was used for protein association networks [44]. KEGG was used for pathway analysis and construction [45]. MoMo was used to discover phosphorylation sequence motifs [46]. Netphos [47] and NetworKIN [48] algorithms were used in some cases to predict phosphorylation sites in terms of kinases and phosphobinding domains. Biorender (BioRender.com, accessed on 3 March 2021) was used for the pathway illustration.

## 3. Results and Discussion

### 3.1. About One Fifth of A. nidulans Predicted Proteins Are Phosphorylated during Growth

To determine phosphoproteomic changes in response to H_2_O_2_, we grew *A. nidulans* mycelia in liquid culture for 12 h, which corresponds to a phase of active hyphal growth [49], and then samples were treated or not with 10 mM H_2_O_2_ for 10 more min. Triplicate biological samples from each condition were processed for phosphoproteomic analysis. These conditions were based on previous results showing that this H_2_O_2_ treatment induces the phosphorylation of the stress MAPK SakA [22,25] and its translocation to the nucleus, where it physically interacts with transcription factor AtfA [33], and other proteins [22] as well as the induction of *catB* gene expression and catalase B activity [24,27,28,33]. Although non-lethal [21], the H_2_O_2_ concentration used in these experiments was relatively high. However, the actual H_2_O_2_ intracellular concentration must be much lower, considering that H_2_O_2_ is efficiently decomposed by the activity of the catalases [24,50], peroxidases, and peroxiredoxins [26,27] expressed under our experimental conditions. Particularly relevant is the fact that part of catalase B is detected at the hyphal cell-wall [51]. As a reference, a 500-fold decreasing gradient from extracellular to intracellular H_2_O_2_ concentration has been estimated in blood plasma [52,53]. More importantly, part of the pathways we found to be affected by H_2_O_2_ (i.e., activation of MAPK, TORC2, and MetR pathways) are involved in the response to other types of physiological stresses such as nutrient starvation.

Most phosphosites considered in our analysis were detected in all three biological replicates. As a rule, a phosphosite was considered only when present in at least two of these replicates. MS/MS based peptide and protein identifications were filtered with Scaffold to obtain phosphorylated peptides. After this, peptide identifications were accepted only if they could be established at ≥90.0% probability by the Peptide Prophet algorithm [42]. Phosphosite identification was established using a FDR of 1.0%, based on a decoy database. With these parameters, the analysis of samples not treated with H_2_O_2_ identified a total of 1964 proteins phosphorylated at one or more sites (Supplementary File S1). This number represents 18.3% of the ORFs predicted by the *Aspergillus* genome database [54], and is larger than the number of *A. nidulans* phosphoproteins identified in other recent studies. For example, 555 phosphoproteins were identified exploring MpkA functions [36], 554 in a study aimed at defining PKA-dependent phosphorylation [37], and 647 in a general survey [38].

We carried out a Gene Ontology [55] and KEGG [56] enrichment analysis to classify the phosphoproteins identified in this work, according to their general function and localization. The three major enriched terms based on biological processes corresponded to intracellular signal transduction, protein transport, and mRNA processing. These are different from the major terms reported for the entire *A*. *nidulans* genome database (AspGD), which correspond to the regulation of biological processes, protein transport, and organelle organization [54]. Regarding molecular function, ATP binding, RNA binding, and protein serine/threonine kinase activity were the three major terms in our phosphoproteome, whereas hydrolase activity, oxidoreductase activity, and transferase activity are the major terms in AspGD. In terms of cellular component analysis, most phosphoproteins we identified are predicted to be localized in the cytoplasm, cell septum and nucleus, while membrane, nucleus, and mitochondrion are the corresponding terms in AspGD. Our KEGG enrichment analysis showed that many of the phosphoproteins detected were involved in MAPK signaling, autophagy, and RNA metabolism.

Overall, these results indicate that under growing conditions, a large proportion of *A. nidulans* phosphoproteome corresponds to proteins involved in cell signal transduction.

### 3.2. H_2_O_2_ Induces the Activation of the SakA/MpkC Stress MAPK Pathway

**Figure 1:**
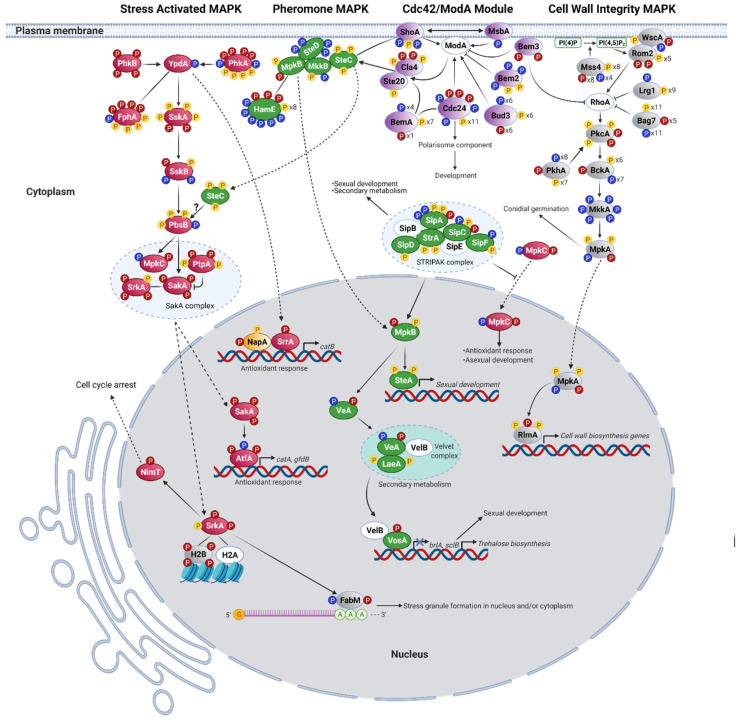
H_2_O_2_ induces the activation of stress (SakA/MpkC), pheromone (MpkB), and cell wall integrity (MpkA) MAPK signaling pathways. H_2_O_2_ induced the phosphorylation of nearly all components of the stress SakA/MpkC MAPK pathway (indicated in magenta). Histidine kinases PhkA, PhkB, and FphA, phosphotransfer protein YpdA, and response regulators SskA and SrrA are components of a stress sensing phosphorelay system. SskA transmits stress signals to the MAPK module composed by MAPKKK SskB, MAPKK PbsB and MAPKs SakA, and MpkC. H_2_O_2_ induced SakA activation through phosphorylation of its TGY activation loop. SakA, MpkC, kinase SrkA, and phosphatase PtpA show physical interaction. Very likely, SrkA and transcription factor AtfA are SakA (and possibly MpkC) substrates. Phosphatase NimT, mRNA-binding protein FabM, and Histone H2B are proposed as SrkA substrates, based on previous physical interaction results. Transcription factors SrrA and NapA, directly involved in the antioxidant response, presented H_2_O_2_-specific phosphorylation. H_2_O_2_ also affected the phosphorylation of multiple components of the pheromone MpkB (indicated in green) and cell wall integrity MpkA (indicated in gray) pathways and the corresponding MAPKs were activated by H_2_O_2_. The pheromone pathway, composed by kinases SteC, MkkB, and MpkB, and by SteD adaptor and HamE scaffold proteins, forms a cytoplasmic pentameric complex. The phosphatase and kinase STRIPAK complex regulates MpkB and MpkC phosphorylation, development, and secondary metabolism. Activated MpkB is translocated to the nucleus, where it could phosphorylate VeA and interact with transcription factor SteA. VeA, VelB, and LaeA methyltransferase form a complex. In the MpkA pathway, mechanosensor WscA transduces the stress signal to GTPase RhoA, which activates protein kinase C PkcA, triggering the MAPK cascade composed by BckA, MkkA, and MpkA. As a critical regulator of cell physiology, RhoA is regulated by GEF Rom2 (AN4719) and GAPs Lrg1 (AN7576), Bem3 (AN5787), and Bag7 (AN11994). Rom2 binds to PI(4,5)P2, generated by Mss4 (AN2766), to activate RhoA. PkcA is also activated by the kinase PkhA. Once activated, MpkA phosphorylates transcription factor RlmA, required for the expression of cell wall biosynthesis genes. The Cdc42 Rho GTPase ModA module (indicated in purple) regulates effector proteins that control the cytoskeleton, membrane-trafficking, and polar growth, and is linked to MAPK regulation. Scaffold protein BemA (AN7030) is critical to localize GEF protein Cdc24 (AN5592), the source of active Cdc42. Bud3 is another ModA GEF and Cla4 (AN8836) and Ste20 (AN2067) PAK kinases are ModA effectors. Membrane proteins Sho1 and MsbA, which were dephosphorylated in H_2_O_2_, could also mediate protein interactions linked to the activation of all three MAPK pathways. H_2_O_2_ affected the phosphorylation of Cdc24, BemA, GAPS Bem2 (AN3746) and Bem3 (AN5787), GEF Bud3, and kinase Cla4. Protein names correspond to *S. cerevisiae*, except when *A. nidulans* protein has been described. Proteins in white background were not detected to be phosphorylated. Yellow P circles indicate phosphosites not affected by H_2_O_2_. Blue phosphosites were dephosphorylated in H_2_O_2_ and red phosphosites correspond to H_2_O_2_-specific phosphosites. Plasma membrane, cytoplasm, and nuclear compartments are indicated. Continuous arrowhead lines indicate positive interaction, T-lines indicate negative interaction, continuous lines indicate interaction, and dotted arrows indicate protein translocation. Relevant references are indicated in the corresponding sections. Created with BioRender.com (accessed on 3 March 2021).

Our results show that H_2_O_2_ induces phosphorylation changes in specific signal transduction pathways. MAP kinases are regulated by phosphorylation at both the threonine and tyrosine of the conserved TXY motif within the activation loop [57]. As indicated before, we used phospho-specific antibodies to show that different types of stress including H_2_O_2_ treatment induce phosphorylation of the MAPK SakA TGY activation loop at T and Y residues [25,33]. Our phosphoproteomic results confirmed this by showing that while SakA was dephosphorylated in the absence of H_2_O_2_, it became phosphorylated at the T171 and Y173 residues, and a novel phosphosite was detected at T176 in the presence of H_2_O_2_ (Figure 1, Table 1). This result validates our experimental design and supports the physiological significance of our phosphorylation analysis. To map the different pathways involved in H_2_O_2_ signaling, we searched the KEGG database for the *A. nidulans* homologs of *Saccharomyces cerevisiae* stress (Hog1), pheromone (Fus3), and cell-wall integrity (Slt1) MAPK pathways and identified their phosphorylation changes in response to H_2_O_2_ (Figure 1, Table 1, Table 2 and Table 3).

The *S. cerevisiae* Hog1 MAPK pathway is mainly involved in osmotic stress sensing. However, its requirement for oxidative stress resistance has also been reported [58]. We detected multiple phosphosites, distributed in different proteins of the Hog1 homologous SakA/MpkC pathway (Table 1). These results, integrated with what is known about the SakA/MpkC pathway in *A. nidulans* and other filamentous fungi, are shown in Figure 1. A histidine-aspartate phosphorelay system that includes 15 histidine kinases, most of them uncharacterized, the essential phosphotransfer protein YpdA, and response regulator SskA has been involved in the activation of stress MAPKs SakA and MpkC [28,33,59]. Although histidine-aspartate phosphorylation is not detectable in our analysis, we identified serine/threonine phosphorylation changes in some of these components in response to H_2_O_2_. A single phosphosite was detected in YpdA [28], which was lost after H_2_O_2_ treatment. The sensor histidine kinases that would transmit H_2_O_2_ signaling to YpdA are not known. This is mediated by histidine kinases Mak2 and Mak3, and indirectly by Mak1, in *Schizosaccharomyces pombe* [59]. Interestingly, we detected four H_2_O_2_-specific phosphosites in Mak1 homolog PhkB and Mak2/Mak3 homolog PhkA. Likewise, four H_2_O_2_-specific phosphosites were detected in histidine kinase FphA, a phytochrome that signals through the SakA pathway [60]. Downstream of YpdA, response regulator SskA contained two phosphosites present only in H_2_O_2_, five that remained unchanged between untreated and treated samples and one that was dephosphorylated in H_2_O_2_. SskA downstream MAPKKK SskB had one H_2_O_2_-specific phosphosite, two dephosphorylated in H_2_O_2_ and one unchanged, while one of four phosphosites detected in MAPKK PbsB was removed after H_2_O_2_ treatment.

Downstream of PbsB, *A. nidulans* and other Aspergilli contain the two homologous stress MAP kinases SakA and MpkC [23,25]. After H_2_O_2_ treatment, SakA was phosphorylated at T171, Y173, and T176, while MpkC lost T176 and acquired Y173. Such inverse phosphorylation of T176 in both MAPKs suggests a physiological relevance that could explain the inverse role that both MAPKs play in response to H_2_O_2_, where SakA is necessary for H_2_O_2_ resistance, while MpkC enhances H_2_O_2_ sensitivity [23]. In any case, H_2_O_2_ affects SakA and MpkC T176 phosphorylation in opposite ways by an unknown kinase, different from the MAPKK PbsB. A NetPhos analysis predicts that a Ca^2+^/Calmodulin kinase (CaM-II) might be responsible for SakA and MpkC T176 phosphorylation. Consistent with this, CaMKII is required for H_2_O_2_-induced phosphorylation of p38 in endothelial cells [61]. Notably, CaM-II kinases CmkA (S376) and CmkC (S140) showed H_2_O_2_-specific phosphorylation, suggesting that they might be involved in H_2_O_2_ sensing and be potentially linked to the SakA/MpkC pathway (Table 1).

Although not regulated by calcium, the SrkA kinase also belongs to the CaM-II kinase family. SrkA is proposed as a SakA substrate because both kinases show physical interaction, and SakA is required for SrkA nuclear localization in response to H_2_O_2_ [22]. In response to osmotic stress, *S. pombe* stress MAPK Sty1 phosphorylates Srk1 at T463, inducing its kinase activity and nuclear translocation, where Srk1 phosphorylates phosphatase Cdc25. Once phosphorylated, Cdc25 interaction with Rad24 (a 14-3-3 protein) promotes its nuclear exclusion, resulting in cell cycle arrest [62]. Our results show four phosphosites in SrkA, with three of them (S31, T412, and T413) being H_2_O_2_-specific. Srk1 T463 corresponds to SakA T413, which NetPhos identifies as a putative substrate of SakA orthologous kinase p38. This supports the role of SakA in SrkA phosphorylation in response to H_2_O_2_ and suggests that SrkA integrates inputs from additional H_2_O_2_-sensitive phosphorylation pathways. A single H_2_O_2_-specific phosphosite was detected in phosphatase NimT, the ortholog of *S. pombe* Cdc25, while 14-3-3 protein AN5744 single phosphosite S211 was dephosphorylated in H_2_O_2_. By analogy with *S. pombe* osmotic response, we propose that *A. nidulans* H_2_O_2_ response involves the sequential phosphorylation of SakA, SrkA, and NimT (Figure 1), promoting NimT interaction with a 14-3-3 protein, its nuclear exclusion, and the arrest of the cell-cycle.

SakA and SrkA physically interact with other proteins including histone H2B (AN3469, the ortholog of *S. cerevisiae* HTB2) [22]. We detected four H2B phosphosites (S11, T12, S137, and S138) present only in H_2_O_2_, and Networkin predicts Ste20 and Pkc1 as the kinases responsible for S137 and S138 phosphorylation, respectively. Notably, previous studies in yeast have shown that Ste20 kinase phosphorylates HTB2 S10 during H_2_O_2_-induced apoptosis. Moreover, phosphonull HTB2 S10A mutants are resistant to H_2_O_2_-induced apoptosis, while phosphomimetic HTB2 S10E mutants induce apoptosis [63]. These results suggest an important role for H2B in responding to H_2_O_2_ as well as its possible regulation by kinases SakA, SrkA, Ste20, and PKC.

The poly-A mRNA binding protein FabM, involved in asexual development [64] and stress granule formation [65] in *A. nidulans*, interacts with SakA and SrkA in response to H_2_O_2_ [22]. Three phosphosites were detected in FabM, two of them (T368, S376) being H_2_O_2_-specific. According to NetPhos, FabM T368 would be phosphorylated by p38, supporting a role for SakA and/or MpkC in FabM phosphorylation. In addition to FabM, the phosphorylation of other putative mRNA-binding proteins was affected by H_2_O_2_ (Table S1), suggesting that some of them might also be involved in the formation of stress granules or other membraneless compartments called molecular condensates. RNA-binding proteins are critical for the formation of some of these condensates, which are formed by a process in which a homogeneous liquid solution of macromolecular components separates into two distinct phases, one enriched for the macromolecules and another depleted of the same macromolecules. Recently, phase separation has been considered to underlie many important biological processes [66]. Seven phosphosites were identified in yeast Pbp1 (ataxin-2) homolog AN1325, from which four were dephosphorylated and one (S267) phosphorylated in H_2_O_2_. Pbp1 interacts with FabM homolog Pab1 to regulate poly(A) tail synthesis [67], and also forms an intracellular condensate required for the inhibition of TORC1 signaling during respiratory growth [68]. Yeast Whi3, another RNA-binding protein involved in stress granule formation [69], is similar to AN7700. Notably, *Ashbya gossypii* Whi3 regulates cell cycle and cell polarity by forming condensate-like structures, and the phosphorylation of individual residues provides specificity to different condensates [70]. In H_2_O_2_, AN7700 was found dephosphorylated at two sites and phosphorylated at T216. These results suggest that by regulating the phosphorylation of these RNA-binding proteins through SakA/MpkC and/or other kinase pathways, H_2_O_2_ might regulate the formation of stress granules and other ribonucleoprotein condensates involved in growth and stress responses. In addition, H_2_O_2_ can regulate liquid–liquid phase separation by mediating the reversible oxidation of proteins like Pbp1, as it occurs in *S. cerevisiae* [71].

Transcription factor AtfA is proposed as a SakA substrate based on mutant epistatic analysis and the fact that both proteins show nuclear physical interaction in response to H_2_O_2_ [33]. *S. pombe* SakA homolog Sty1/Spc1 phosphorylates AtfA homolog Atf1 at the serine in PLSP (S172) motif, and this regulates its stability and function [72]. We detected three AtfA phosphosites; one absent after H_2_O_2_ treatment (T159) and two (S132 and S136) being H_2_O_2_-specific (Table 1). A protein alignment of AtfA and Atf1 shows that the PLSP motif is conserved in both proteins and that the serine in this motif corresponds to AtfA S136. These results support AtfA S136 phosphorylation by SakA, as a response to H_2_O_2_.

The phosphatases responsible for SakA dephosphorylation are not known in *A. nidulans*. However, phosphatase PtpA (AN6982) shows constitutive physical interaction with SakA [22] and is the ortholog of *S. pombe* phosphatase pyp1, involved in the negative regulation of the stress MAPK Sty1/Spc1 [73]. We detected four phosphosites in PtpA, two invariable and two phosphorylated after H_2_O_2_ treatment (Table 1, Figure 1). This suggests that H_2_O_2_ might regulate not only SakA phosphorylation, but also its dephosphorylation, as SakA constitutive phosphorylation is lethal [33]. In addition, H_2_O_2_ affected the phosphorylation of other phosphatases (Table S2). Five phosphosites were detected in phosphatase PtcD (AN0914), four of which were H_2_O_2_-specific. Ptc4, *S. pombe* PtcD ortholog, is a mitochondrial phosphatase that interacts with SakA ortholog Sty1, and its elimination results in prolonged Sty1 activation upon H_2_O_2_ treatment, but not under other types of stress [74]. Ten phosphosites were detected in phosphatase CdcA (AN5057); two dephosphorylated in H_2_O_2_ and two H_2_O_2_-specific. *S. cerevisiae* CdcA homolog Cdc14 is essential to regulate Cdk1/Cdc28 (NimX in *A. nidulans*) kinase activity and mitotic exit [75]. Phosphatase PsrA (AN10077) presented seven phosphosites; six dephosphorylated in H_2_O_2_ and one (S151) H_2_O_2_-specific. PsrA is required for conidia germination on glucose media [76]. *S. cerevisiae* PsrA homolog Psr2 inhibits TORC1 signaling through its interaction with Whi2 (see TOR section). In H_2_O_2_, putative alkaline phosphatase Pho8/AN10563 was dephosphorylated at S150 and phosphorylated on S14. *S. cerevisiae* Pho8 is involved in the regulation of autophagy [77]. Phosphatase DipA controls septa positioning and the phosphorylation-dependent stability of deneddylase DenA/DEN1 [78]. Moreover, DipA associates with early endosomes, depending on the presence of endosome-peroxisome linker protein PxdA, and regulates peroxisome movement and distribution [79]. H_2_O_2_ not only affected DipA phosphorylation, but also the phosphorylation of its substrate DenA (Table S7; see proteosome section) and its potential substrate PxdA. Of the thirteen phosphosites detected in DipA, eight were dephosphorylated and one phosphorylated in H_2_O_2_. Twelve phosphosites were detected in PxdA, four of which were dephosphorylated in H_2_O_2_ (not shown). These results suggest that H_2_O_2_ regulates both proteolysis and peroxisome dynamics by regulating the activation of DipA. Notably, H_2_O_2_ also differentially affected the phosphorylation of both PP2A (protein phosphatase 2A) complex regulatory subunits [80]. The PP2A phosphatase complex plays a conserved role in cell cycle and TOR signaling in eukaryotes (see TOR section).

In summary, our results show that H_2_O_2_ activates the SakA/MpkC pathway and support its role in the phosphorylation of AtfA, SrkA, NimT, FabM, and histone H2B. They also suggest a connection between this pathway and CAM-II kinases CmkA and CmkC in H_2_O_2_ sensing. Moreover, H_2_O_2_ also regulates the phosphorylation of several mRNA-binding proteins and phosphatases, potentially related to this and other MAPK pathways.

### 3.3. H_2_O_2_ in the Activation of the MpkB Pheromone MAPK Pathway

The pheromone pathway in *A. nidulans* regulates not only development, but also secondary metabolism and has been involved in cell wall and oxidative stress responses [81]. It is composed by MAPKKK SteC (Ste11 in yeast), MAPKK MkkB (Ste7), and MAPK MpkB (Fus3) as well as the adaptor SteD (Ste50) and the scaffold protein HamE [81], all of which form a pentameric complex [82,83]. G-protein coupled receptors (GPCR) GprA and GprB, related to yeast pheromone receptors Ste2 and Ste3, would presumably signal to the MpkB pathway, modulated by heterotrimeric G-proteins [84]. *A. nidulans* contains G-alpha subunits FadA, GanA, and GanB. FadA and GanB signaling is mediated by the cAMP/PKA pathway to regulate development and secondary metabolism [85,86] and conidial germination [87], while GanA functions are less clear. We detected a unique phosphosite in GanB, which was dephosphorylated in H_2_O_2_ (Table 2). As shown in Table 2 and Figure 1, four phosphosites detected in SteD were also dephosphorylated in H_2_O_2_. SteC contained three invariable phosphosites, while one of the two phosphosites detected in MkkB was dephosphorylated H_2_O_2_. MkkB and MpkB are bound by scaffold protein HamE, which might also have pheromone-independent functions [83]. Eight phosphosites have been previously identified in HamE [82]. We confirmed two of these phosphosites (S425 and S973) and detected a more complex phosphorylation pattern for HamE, with a total of 17 phosphosites: six dephosphorylated in H_2_O_2_ and three H_2_O_2_-specific. MpkB was found phosphorylated at T182 (only in H_2_O_2_) and Y184 (with and without H_2_O_2_). This indicates that MpkB is activated by H_2_O_2_, as phosphorylation of both residues in the TEY activation loop is required for activity in all members of this MAPK family [88].

The striatin-interacting phosphatase and kinase (STRIPAK) complex regulates many cellular processes including signal transduction and development [89]. *A. nidulans* STRIPAK complex, composed of StrA, SipA, SipB, SipC, SipD, SipE, and SipF subunits, is required for MpkB phosphorylation and regulates resistance to oxidative stress, development, and secondary metabolism [90]. We detected five of the seven STRIPAK subunits as phosphoproteins, but only SipA (S337) and SipC (T84) contained H_2_O_2_-specific phosphosites (Table 2). Notably, *sipA* deletion results in increased resistance to H_2_O_2_, while *sipC* deletion causes increased sensitivity to H_2_O_2_. Moreover, SipA interacts with the rest of the complex only during development and seems to play an inhibitory role [90]. This suggests H_2_O_2_ might regulate STRIPAK complex activity by modulating the phosphorylation of SipA and SipC subunits.

Phosphorylated MpkB regulates secondary metabolism and development in *A. nidulans* and other fungi [81]. These two processes are closely related in filamentous fungi, in part because some products of secondary metabolism function as signaling molecules that initiate asexual development [91,92,93]. The synthesis of many secondary metabolites depends on gene clusters globally regulated by putative methyltransferase LaeA [94]. LaeA is part of a methyltransferase complex that includes proteins VeA and VelB, which plays critical roles in secondary metabolism and development [95]. In *A. flavus*, VosA [96] and VeA [97] have also been involved in the oxidative stress response. Notably, MpkB phosphorylates VeA in vitro [81]. We detected H_2_O_2_-specific phosphosites in VeA (S374) and VosA (T247) and two invariable phosphosites in LaeA (S39 and S70) and SteA (S550 and T552). In addition, several enzymes directly involved in the biosynthesis of secondary metabolites such as fatty acid synthases (FAS) and polyketide synthases (PKS) were detected as phosphoproteins (Table S3). Several lines of evidence indicate that oxidative stress regulates fungal secondary metabolism at the transcriptional level [98,99]. However, little is known about the regulation of secondary metabolism enzymes by phosphorylation. We detected that StcJ, a PKS required for sterigmatocystin biosynthesis [100], had two unique H_2_O_2_-specific phosphosites. AN7873, predicted as a member of a secondary metabolism gene cluster [101], contained six unique H_2_O_2_-specific phosphosites. This protein is the ortholog of the *S. cerevisiae* Fas1 fatty acid synthetase beta subunit, involved in the synthesis of long-chain saturated fatty acids. Notably, *fas1* downregulation increases the resistance to H_2_O_2_ [102]. AN3612, a putative PKS [103], had three phosphosites only in H_2_O_2_.

Asexual development (conidiation) in *A. nidulans* depends on the expression of the master gene *brlA* [104,105] and of several genes required for the proper expression of *brlA* including *flbC*, *tmpA*, and *stuA* [12,93,106,107]. Downstream of *brlA*, genes like *apsA*, *apsB*, *wetA*, and *dopey* [105,106,107,108] are required for proper conidia development. All the proteins encoded by these genes were detected as phosphoproteins (Supplementary file) and StuA was detected as a H_2_O_2_-specific phosphoprotein (Table S5).

The ImeB kinase, conserved form fungi to animals, is related to MpkB function in *A. nidulans* as it is required for light-mediated inhibition of sexual development and for mycotoxin production in a VeA-dependent fashion. ImeB contains a TXY motif (TTY) of the type found in MAP kinases, and the substitution of any of these three amino acids produced inactive versions of this kinase [109]. Moreover, the phosphorylation of T and Y in the TXY motif was shown to be essential for the activity of ImeB ortholog Crk1 in *Ustilago maydis* [110]. We detected eight phosphosites in ImeB (Table 2); three dephosphorylated and four phosphorylated in H_2_O_2_. Notably, we found all three TTY residues phosphorylated; H_2_O_2_ induced the dephosphorylation of T211 and the phosphorylation of T210, while Y212 phosphorylation was not affected by H_2_O_2_. This suggests that H_2_O_2_ regulates sexual development and secondary metabolism by activating MpkB and by modulating ImeB activity, possibly through the differential phosphorylation of its TTY motif.

### 3.4. H_2_O_2_ in the Regulation of RhoA and the Activation of the MpkA Cell Wall Integrity Pathway (CWI)

The fungal cell wall, composed mainly of chitin and glucans, needs to be remodeled during polar growth, cell differentiation, and in response to environmental stress. Although SakA, MpkC [23], and MpkB [111] have been involved in resistance to cell wall stress in *A. nidulans*, MpkA is the MAPK generally associated with cell wall maintenance and repair pathways, which are similar in yeast [112] and *A. nidulans* [113]. In these organisms, cell surface mechanosensors transduce stress signals to RhoA GTPase, which in turns activates protein kinase C PkcA, triggering the activation of the MAPK cascade composed of BckA, MkkA, and MpkA. MpkA phosphorylates several proteins including transcription factor RlmA (Figure 1), which is involved in the expression of genes related to ∝-glucan biosynthesis [113]. Rho1 is a major integrator of cell physiology in yeast and other fungi. In yeast, it is activated by cell cycle effectors and regulates Pkc1 and Fks1/2 enzymes involved in cell wall polymer biosynthesis, and other effectors involved in actin organization and exocytosis (see [112] for a review). This explains that Rho1 activity is highly regulated by three GEFs (Rom1, Rom2 and Tus1), four GAPs (Bem2, Sac7, Lrg1 and Bag7,) and phosphoinositide pathway enzymes Stt4 and Mss4. Sst4 and Mss4 are responsible for the production of phosphoinositides PI(4)P and PI(4,5)P2, respectively. PI(4,5)P2 is critical to recruit Rom2 to the plasma membrane to activate Rho1 [112]. As discussed further below, PI(4,5)P2 also plays important roles in the regulation of TORC2 and other signaling pathways [114].

Our results show that the phosphorylation patterns of some of these critical regulators of RhoA and CWI pathway activity are affected by H_2_O_2_ (Figure 1, Table 2, Table 3 and Table 4). Although we did not detect RhoA as a phosphoprotein, its GEF Rom2, also linked to RhoA function in *A. fumigatus* [115], contained seven phosphosites; two found only in H_2_O_2_. Ten phosphosites were detected in RhoA GAP Lrg1; one dephosphorylated in H_2_O_2_. Two unique H_2_O_2_-specific phosphosites were detected in RhoA GAP Bem3 ortholog AN5787. Notably, 27 total phosphosites were detected in RhoA GAP Bag7; 11 dephosphorylated in H_2_O_2_ and five H_2_O_2_-specific. We propose that these H_2_O_2_-induced phosphorylation changes in Rom2, Bag7, and Bem3 result in RhoA activation.

**Figure 2:**
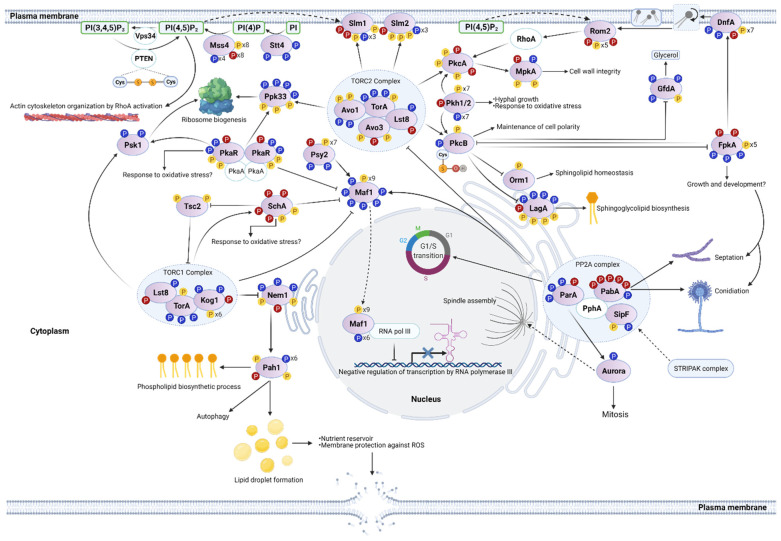
H_2_O_2_ induces major phosphorylation changes in critical components of phosphoinositide, TOR, and MpkB signaling. Phsophoinositide kinase Stt4 (AN4278) synthesize phosphoinositide PI(4)P, which is utilized by Mss4 (AN2766) to produce PI(4,5)P2 (indicated in green rectangles). Lipid kinase Vps34 (AN4709) synthesize PI(3,4,5)P3, a substrate of phosphatase PTEN (AN10780) that transforms it back to PI(4,5)P2. Vps34 and PTEN were not detected as phosphoproteins. We propose that Stt4 and Mss4 H_2_O_2_-specific phosphorylation changes result in their activation, at the same time that H_2_O_2_ oxidation of PTEN conserved cysteines inactivates this phosphatase as it occurs in animal cells. This would enhance PI(3,4,5)P3 levels, which can stimulate Ca^2+^ release from intracellular stores (not shown). High PI(4,5)P2 levels target Rho1 GEF Rom2 (AN4719) to the plasma membrane, resulting in the sequential activation of GTPase Rho1 (AN5740) and CWI MAPK MpkA (AN5666). PI(4,5)P2 and other phosphoinositides recruit and/or activate diverse effector proteins containing PH, FYVE, or PX domains including the targeting of PH domain proteins Slm1 (AN5671) and Slm2 (AN4171) to the plasma membrane. Slm1 and Slm2, which are dephosphorylated in H_2_O_2_ but also acquire H_2_O_2_-specific phosphosites, would be required for TORC2-dependent phosphorylation of PkcB (Ypk1), as it occurs in yeast. TORC2 membrane targeting and perhaps the H_2_O_2_-specific phosphorylation of TORC2-specific component Avo3 (AN10756) would result in activation of its kinase activity. AGC kinases PkcA (Pkc1) and PkcB (Ypk1) are the main targets of both Pkh1/2 (AN3110) and TORC2. PkcA contains two H_2_O_2_-specific phosphosites, suggesting that H_2_O_2_ activates both TORC2 and PkcA, which in turn activates MpkA. Pkh1 essential phosphorylation site in PkcB is dephosphorylated in H_2_O_2_ indicating its inactivation by H_2_O_2_, perhaps mediated by the oxidation of a conserved cysteine. The dephosphorylation of several phosphosites in putative PkcB substrates LagA and FpkA is consistent with this. We propose that as in *S. cerevisiae*, PkcB negatively regulates both LagA and FpkA and that PkcB inactivation by H_2_O_2_ results in an increased biosynthesis of sphingoglycolipids by ceramide synthase complex subunit LagA and a specific phosphorylation of flipase DnfA by kinase FpkA. DnfA regulates membrane fluidity, which together with PI(4,5)P2, increase Rom2 targeting to the plasma membrane, resulting in the activation of the cell wall integrity pathway. Likewise, PkcB putative substrate NAD+ dependent glycerol-3-phosphate dehydrogenase GfdA is found dephosphorylated in H_2_O_2_, consistent with an increased biosynthesis of glycerol in H_2_O_2_, as it occurs in *S. cerevisiae*. In addition, PkcB putative substrate Orm1 (AN1933) does not show phosphorylation changes in H_2_O_2_, suggesting its activation and a higher sphingolipid biosynthesis. Other putative TORC2 substrates, the kinases Ppk33 (AN7537) and Psk1 (AN4980), are also regulated by PkaA. Ppk33 and Psk1 regulate ribosomal biogenesis and growth. The dephosphorylation of these two kinases in H_2_O_2_ suggests the inhibition of ribosome biosynthesis by H_2_O_2_. Consistent with this, the transcription factor Maf1 (AN7681) was dephosphorylated in H_2_O_2_, which would result in its nuclear localization, the negative regulation of transcription by RNA polymerase III, and the inhibition of ribosome biogenesis. Maf1 phosphorylation depends on kinase SchA (AN4238) and phosphatase Psy2 (AN7750), which were phosphorylated in H_2_O_2_ as well as on TORC1 activity. This suggests that H_2_O_2_ induces the activation of Psy2 and the inactivation of SchA and TORC1. TORC1 inactivation would result in phosphatase Nem1 (AN1343) activation and the consequent dephosphorylation of phosphatidate phosphatase Pah1 (AN0802). Pah1 dephosphorylation stimulates the biosynthesis of phospholipids and lipid droplets as well as the process of autophagy. Phosphatase complex PP2A regulates growth and cell cycle by regulating the dephosphorylation of Maf1, negatively regulating TORC2 and the phosphorylation of Aurora kinase (AN5815). H_2_O_2_ induced the dephosphorylation and phosphorylation of alternative regulatory subunits ParA and PabA, respectively. ParA and PabA determine PP2A substrate specificity. Aurora kinase single phosphosite was dephosphorylated in H_2_O_2_. Protein names correspond to *S. cerevisiae*, except when the *A. nidulans* protein has been described. Color codes for phosphorylation sites and interaction lines are as in Figure 1. Relevant references are indicated in the corresponding sections. Created with BioRender.com (accessed on 3 March 2021).

As shown in Table 5 and Figure 1 and Figure 2, the ortholog of phosphoinositide kinase Sst4 (AN4278/SstA), required for the biosynthesis of PI(4)P in yeast, contained three phosphosites (Y788, S790, T792) and all were dephosphorylated in H_2_O_2_. Phosphoinositide kinase AN2766 (MssA, Table 5), the ortholog of *S. cerevisiae* PtdIns(4,5)P2 producing kinase Mss4 [116], contained 20 phosphosites; four dephosphorylated and eight phosphorylated in H_2_O_2_. All these phosphosites (amino acids S65–S284) are concentrated in the N-terminal portion of the protein including N-terminal and kinase domains. Part of the *N. crassa* MSS-4 N-terminal domain is critical for its correct membrane association [117], while *S. cerevisiae* Mss4 hyperphosphorylation results in increased plasma membrane association and a higher production of PtdIns(4,5)P2 [114]. Therefore, we propose that the H_2_O_2_-induced phosphorylation changes in thee *A. nidulans* Sst4 and Mss4 homologs result in the activation of the phosphoinositide signaling pathway, leading to the activation of both the RhoA-MpkA and TORC2 signaling pathways (see phosphoinositide/TOR section). Along this line, the phosphorylation of *A. nidulans* phosphoinositide-dependent kinase PkhA (also known as An-ksg1) was also heavily affected by H_2_O_2_, which induced the dephosphorylation of eight residues and the phosphorylation of T207 (Figure 1 and Figure 2, Table 3). PkhA [118,119] is the homolog of mammalian PDK1 and *S. cerevisiae* Pkh2 (see TOR section), the second one being involved in Pkc1 phosphorylation and MAPK activation [120], and also being a major TORC2 substrate (TOR section). PkcA, a putative target of PkhA, contained five phosphosites, two of them H_2_O_2_-specific. Fourteen phosphosites were detected in MAPKKK BckA; seven dephosphorylated and one phosphorylated in H_2_O_2_. Somewhat unexpectedly, MkkA six phosphosites were found to be dephosphorylated in H_2_O_2_. Nevertheless, the MAPK MpkA was detected active (phosphorylated at T100 and Y102) with and without H_2_O_2_. Notably, we detected additional phosphosites Y98 and T105, which in H_2_O_2_ were dephosphorylated and phosphorylated, respectively. A recent report shows that yeast MpkA homolog Slt2 contains two phosphosites outside the TEY motif, one of which (T195) is also critical for the activity of this MAPK [121]. As MpkA T105 corresponds to Slt2 T195, our results suggests that the H_2_O_2_-induced phosphorylation of MpkA at T105 contributes to its activation. Downstream of MpkA, the transcription factor RlmA contained two invariant and one (T315) H_2_O_2_-specific phosphosites.

In summary, our results are consistent with a model in which H_2_O_2_ activates phosphoinositide signaling (discussed in more detail in the next section) with the consequent activation of RhoA and the downstream CWI pathway to regulate cell wall polymer biosynthesis, actin organization, exocytosis, and the cell cycle.

### 3.5. Msb2-Sho1 and the Cdc42 Module as Integrators of MAPK Signaling in Response to H_2_O_2_

Cdc42 GTPases are conserved regulators of cell polarity, cell cycle, and morphogenesis in eukaryotic cells [122,123,124]. In *A. nidulans*, Cdc42 homologue ModA plays a major role in polarity establishment [125]. In *S. cerevisiae*, the active GTP-bound conformation of Cdc42 binds to and activates PAK kinases Cla4 and Ste20 [122,123], and both kinases can differentially activate the pheromone and osmotic stress Hog1 MAPK pathways [122,126]. In addition to Ste20 and Cla4, Cdc42 binds the Ste11-Ste50 (SteC-SteD) complex to bring activated Ste20/Cla4 to their substrate Ste11. The membrane protein Sho1 binds active Ste11 and MAPKK PbsB to activate the Hog1 pathway [126], and mucin Msb2 and Sho1 collaborate to activate Hog1 in response to osmostress, as has also been proposed in *A. fumigatus* [127]. Other critical regulators of Cdc42 activity are its GEF protein Cdc24 and the scaffold protein Bem1 (BemA), which are critical to localize Cdc24, the actual source of active Cdc42, to the growing front of yeast and filamentous fungi to organize cell polarity [128]. Bud3, another Cdc42 GEF protein, is part of a transient axial landmark complex that localizes at the mother-bud neck during septin ring formation and cell division [129]. Bem2 and Bem3 are Cdc42 GAP proteins that are found hyperphosphorylated at bud emerging points, which appears to inhibit their GAP activity [130]. Bem3 contains contiguous PX and PH domains that appear to collaborate in phosphoinositide binding and membrane interactions [131].

While we did not detect Cdc42 (ModA) as a phosphoprotein, the phosphorylation pattern of critical regulators of its activity was notably affected by H_2_O_2_ (Figure 1, Table 4). Indeed, H_2_O_2_ induced the dephosphorylation of ShoA at two sites (T207, S215), while MsbA single phosphosite was dephosphorylated in H_2_O_2_. Two H_2_O_2_-specific phosphosites (T64, T66) were detected in Cla4 and, notably, 17 phosphosites were detected in Cdc24 (AN5592); three dephosphorylated and three phosphorylated in H_2_O_2_. Eighteen phosphosites were found in Bud3 (AN0113); six dephosphorylated and six phosphorylated in H_2_O_2_. Twelve phosphosites were detected in Bem1 scaffold protein homolog BemA; four absent in H_2_O_2_ and one H_2_O_2_-specific. These results suggest that ShoA, MsbA, and ModA integrate different environmental inputs including ROS signals to regulate actin and septin cytoskeletons and polarized secretion via their downstream effectors including PAKs Cla4 and Ste20, MpkB, and possibly SakA MAPKs.

### 3.6. H_2_O_2_ in Phosphoinositide and TOR Signaling

The phosphoinositide family (PIs) includes seven types of phosphorylated phosphatidylinositol, localized in different organelle membranes, which function as lipid signaling molecules by recruiting and/or activating diverse effector proteins. Although PI(5)P and PI(3,4)P2 have not been detected in fungi, PI(4,5)P2 has been involved in the regulation of the actin cytoskeleton, endocytosis, and Rho-Pkc1 signaling cascade in yeast [132]. The levels of the different PIs depend on specific kinases, phospholipases, and phosphatases. PI(4,5)P2, the most abundant PI is synthesized in *S. cerevisiae* by Mss4 phosphatidylinositol 4-phosphate 5-kinase or by Tep1, a homologue of mammalian PTEN phosphatase [133]. By catalyzing the dephosphorylation of PI(3,4,5)P3 to produce PI(4,5)P2, PTEN can regulate the levels of both PIs. In fact, the elimination of PTEN homolog in *S. pombe* results in the accumulation of its substrate PI (3,4,5)P3 [133]. The hydrolysis of PI(4,5)P2 by phospholipase C produces inositol 1,4,5-triphosphate (InsP3) and diacylglycerol (DAG). InsP3 can directly stimulate Ca^2+^ release from intracellular stores [134], therefore linking phosphoinositide and Ca^2+^ signaling.

As indicated previously (RhoA and MpkA section), our results show that H_2_O_2_ induced major changes in the phosphorylation of phosphoinositide kinases Stt4 and Mss4 (Figure 1 and Figure 2, Table 5). In addition, two phosphosites (S94, S672) were detected in inositol polyphosphate kinase VlpA (AN5797), which generates inositol pyrophosphates and is required for proper growth and microtuble dynamics [135], and one (S94) was dephosphorylated in H_2_O_2_. Moreover, H_2_O_2_ induced phosphorylation changes in putative phosphatidyl transfer protein AN3709, phosphatidyl kinase regulator AN5857, putative phospholipases C (AN10413) and D (AN2947, PlcA), and phosphatidylinositol phosphatases AN7745, AN8053, and AN0925 (Table 5). Although we did not detect the *A. nidulans* PTEN phosphatase ortholog AN10780 as a phosphoprotein, numerous studies have demonstrated that the catalytic activity of PTEN is inhibited by H_2_O_2_ [136,137]. In animal cells, PTEN oxidative modification contributes to increase PI(3,4,5)P3 levels, resulting in activation of PI3K/AKT signaling and oxidative stress-induced cell death protection. PTEN can also dephosphorylate itself and other protein substrates. The essential active Cys124 residue of human PTEN, surrounded by three basic amino acid residues in the active site pocket, is readily oxidized by forming an intramolecular disulfide with Cys71 [137]. This reversible H_2_O_2_-mediated oxidation of PTEN is reduced mainly by thioredoxin. The PTEN redox sensitive region including the two cysteines, is highly conserved in AN10780 (Figure 2). This suggests that this phosphatase is inactivated by H_2_O_2_ under our experimental conditions, which would result in increased PI(3,4,5)P3 levels [138].

In addition to regulating the MpkA MAPK pathway, phosphoinositides are critical to recruit and/or activate diverse effector proteins, mainly through FYVE, PH, or PX domains including PAK kinase Cla4, Rho1 GEF Rom2, Cdc42 GAP Bem3 (Figure 1), and TORC2 plasma membrane recruiting and actin regulating proteins Slm1 and Slm2 (Figure 2). H_2_O_2_ affected the phosphorylation of multiple proteins containing FYVE (Table 6), PH (Table 7), or PX (Table 8) domains. The diversity of processes in which these effector proteins are potentially involved would explain the pleiotropic nature of H_2_O_2_ and phosphoinositide signaling. Moreover, there is a close interrelation between the functions of plasma membrane domains known as the MCC/eisosome complex and phosphoinositide signaling. The formation of these plasma membrane protein-organized invaginations is promoted by proteins Pil1 and Lsp1, which contain BAR domains and bind PI(4,5)P2. The MCC/eisosome is considered as a membrane tension sensor complex and has also been involved in morphogenesis, cell wall integrity, and environmental stress resistance (see [139] for a recent review). We detected that the phosphorylation of MCC/eisosome-localized proteins PilA (AN5217), Nce102 (AN7683), PkhA, and Slm2 was affected by H_2_O_2_. PilA contained a unique H_2_O_2_-specific phosphosite (S60), while the Nce102 single phosphosite (S170) was dephosphorylated in H_2_O_2_. Pil1 phosphorylation has been associated with eisosome stability. However, is not clear yet whether such phosphorylation increases or decreases eisosome stability under different growth conditions [139]. It is interesting that flavodoxin-like proteins enriched in MCC/eisosome domains are important for oxidative stress resistance in *Candida albicans* [140].

Overall, our results show that H_2_O_2_ has a major impact in the phosphorylation of multiple proteins involved in phosphoinositide pathways, suggesting that H_2_O_2_ signaling is largely mediated by phosphoinositide signaling, which includes MpkA and TOR kinase signaling.

The target of rapamycin (TOR) is a conserved kinase of the phosphoinositide 3-kinase (PI3K)-related kinase family that actually functions as a serine/threonine kinase. It plays a crucial role in the integration of diverse environmental cues such as nutritional status and stress to regulate cell growth in eukaryotes. The TOR kinase forms multi-subunit TOR complex 1 (TORC1) and TOR complex 2 (TORC2), which show different substrate specificities. These complexes control kinases of the AGC family, which play essential roles in growth regulation. However, for any activity at all, these AGC kinases must be phosphorylated on a threonine conserved in their activation loop (T-loop) by the 3-phosphoinositide dependent kinase PDK1 [141]. PDK1, itself an AGC kinase, catalyzes its own essential activation loop phosphorylation in *trans* and although considered as constitutively active, other phosphorylation and dimerization events can affect its activity [142]. In contrast to some AGC kinases, PDK1 is essential and present in all eukaryotes. In animal cells, phosphoinositides present at the plasma membrane recruit PDK1 and its substrate kinase Akt, favoring conformational changes that result in Akt phosphorylation. In *S. cerevisiae*, PDK1 orthologs Pkh1 and Pkh2 activate Ypk1 and Ypk2 essential downstream kinases, which have been implicated in the regulation of endocytosis, membrane expansion, and cell wall remodeling [141].

We found that H_2_O_2_ affected the phosphorylation patterns of components of both TOR complexes as well as the phosphorylation of some downstream AGC kinases (Figure 2, Table 9). In contrast to yeast, *A. nidulans* contains a single TOR kinase called TorA, which contained four phosphosites during growth and three of them were dephosphorylated in H_2_O_2_. In the case of TORC1 subunits, H_2_O_2_ induced the dephosphorylation of AN4639 at two sites (S20, S990) and its phosphorylation at S985. AN4639 is the ortholog of *S. cerevisiae* TORC1-specific subunit Kog1 (RAPTOR in humans). Likewise, H_2_O_2_ induced the dephosphorylation of Lst8 ortholog AN1335 at S267 and its phosphorylation at S273. *S. cerevisiae* Lst8 associates with both TORC1 and TORC2 complexes. Consistent with this, mutation of *LST8* produces effects similar to the ones produced by rapamycin, which inhibits TORC1 but not TORC2 as well as cell wall defects mediated by TORC2 [143]. Downstream of TORC1, *S. cerevisiae* Sch9 AGC kinase is phosphorylated by Tor1 under nitrogen rich conditions and required for TORC-mediated regulation of ribosome biogenesis and entry into G0 phase. *A. nidulans* Sch9 ortholog SchA plays redundant functions with protein kinase A (PkaA) during asexual spore germination and participates in carbon catabolite repression [144]. In *A. fumigatus*, *schA* gene expression is dependent on SakA MAPK and Δ*schA* mutants show increased SakA phosphorylation, suggesting a link between TORC1 and the SakA pathway [145]. Of six phosphosites detected in SchA, two were invariable (S240, S769) and four (S227, S233, S359, and S360) were detected only in H_2_O_2_.

Nutrient starvation and TORC1 inactivation promote autophagy. In *S. cerevisiae* both, TORC1 inactivation and phosphatase Nem1 activation result in dephosphorylation and activation of the lipin phosphatidate phosphatase Pah1, which is necessary for autophagy (Figure 2) [146]. Moreover, *pah1* deletion results in H_2_O_2_ hypersensitivity [147] and reduced lipid droplet content [148]. In H_2_O_2_, the Nem1 ortholog AN1343 was phosphorylated at S158 and dephosphorylated at three phosphosites, while Pah1 ortholog AN0802 was phosphorylated at S182 and dephosphorylated at six phosphosites. These results suggest that H_2_O_2_ induces TORC1 inactivation, Nem1 activation, and the dephosphorylation and activation of Pah1 (Figure 2). This, in turn, would lead to an increase in autophagy and lipid droplet biosynthesis. In stressed cells, lipid droplets can maintain energy and redox homeostasis by sequestering toxic lipids and by participating in the maintenance of membrane and organelle homeostasis [149]. Autophagy and lipid droplet utilization are crucial for stressed cell survival [149].

The transcription factor Maf1 is a TORC1-regulated master regulator, essential to modulate transcription in response to changing nutritional and cellular stress conditions. In *S. cerevisiae* and many other eukaryotes, Maf1 plays an important role under adverse growth conditions by repressing RNA polymerase III-mediated gene expression, and the consequent decrease in protein synthesis. Notably, both rapamycin and H_2_O_2_ induce Maf1 dephosphorylation and its nuclear accumulation, in a process that depends on the activities of phosphatase PP2A and thioredoxins Trx1 and Trx2 [150]. Under optimal growth conditions, Maf1 is phosphorylated on consensus PKA sites by both PKA and Sch9 AGC kinases [151]. Although we did not detect *A. nidulans* PKA as a phosphoprotein, PKA regulatory subunit PkaR (AN4987) was found phosphorylated at S53 and dephosphorylated a two phosphosites in H_2_O_2_, suggesting that PkaR might play a role in Maf1 phosphorylation and the oxidative stress response. In agreement with this, conidia from *pkaR* null mutants are sensitive to H_2_O_2_ in *A. fumigatus* [152]. Consistent with Maf1 regulation in *S. cerevisiae*, we detected that Maf1 ortholog AN7681 was highly phosphorylated (15 phosphosites) during growth (in the absence of H_2_O_2_) and dephosphorylated at six residues in the presence of H_2_O_2_. Phosphatase A7750, a potential candidate to dephosphorylate Maf1 ortholog AN7681, was detected with ten total phosphosites, from which two were dephosphorylated in H_2_O_2_ and one was H_2_O_2_-specific (Table 9, Figure 2).

TORC1 function is also related to the PP2A phosphatase complex in *S. cerevisiae*, where both transduce stress signals to transcription factor Msn2 [153]. Moreover, PP2A phosphatase complex crucial role in cell cycle regulation is conserved from yeast to humans. The *S. cerevisiae* PP2A complex is formed by the two nearly identical catalytic subunits Pph21 and Pph22, scaffold protein Tpd3, and regulatory subunits CDC55 and Rts1, which determine substrate specificity [80]. The absence of Rts1 results in an increased phosphorylation on two residues of Aurora kinase catalytic subunit Ipl1 [154], which plays a critical role in mitosis. In our analysis, we did not detect *A. nidulans* PP2A catalytic subunit PphA. However, we detected five H_2_O_2_-specific phosphosites in CDC55 ortholog PabA (AN1545). In contrast, Rts1 ortholog ParA (AN9467) was dephosphorylated at three phosphosites and phosphorylated on only one site (S147) in H_2_O_2_ (Figure 2), while the only phosphosite (S80) detected in Aurora kinase (AN5815) was dephosphorylated in H_2_O_2_. The differential phosphorylation of PP2A regulatory subunits in H_2_O_2_ suggests that H_2_O_2_ regulates PP2A alternative substrate dephosphorylation. These results are consistent with the fact that in *A. nidulans*, ParA and PabA show opposite functions in septation and common functions during hyphal growth [155].

The TORC2 complex plays a critical role for the survival of human cells to H_2_O_2_, and TORC2-specific regulatory subunit RICTOR is involved in hydrogen peroxide-mediated stimulation of phospho-Akt-S473 levels [156]. RICTOR plays a major role in the assembly and stabilization of the TORC2 complex, while SIN1 determines substrate-binding [157,158]. In *S. cerevisiae* and other fungi, TORC2 is localized at the plasma membrane, where it works as a sensor and master regulator of plasma membrane and cell-wall homeostasis by phosphorylating and activating Ypk1 (and paralogous Ypk2) and Pkc1 [141]. The *S. cerevisiae* TORC2 complex consists of specific subunits Tsc11 (RICTOR ortholog, also known as Avo3), Avo1 (SIN1 ortholog), and TOR common subunit Lst8. We detected three phosphosites in the Tsc11 ortholog AN10756, one of them phosphorylated (Y108) only in H_2_O_2_.

TORC2 activates AGC kinase Ypk1 by phosphorylating multiple sites at its C-terminus. However, as indicated before, Ypk1 phosphorylation at the T-loop (T-504) by PDK kinases Pkh1/2 is essential for its activity [159,160]. Our results show that TorA, *A. nidulans* Avo1 (AN6304), and Ypk1 (PkcB, AN5973) orthologs were all dephosphorylated in the presence of H_2_O_2_ (Table 9, Figure 2). Notably, an alignment of AGC kinases Ypk1, AKT, SGK1, and *A. nidulans* PkcB shows that Ypk1 T504 is part of a well conserved region in all these kinases and that Ypk1 T504 corresponds to PkcB T450, the residue that was dephosphorylated in H_2_O_2_. These results indicate that H_2_O_2_ induces the inactivation of PkcB. In contrast, we did not detect T-loop phosphorylation in other members of the AGC family such as SchA and PkcA, suggesting low phosphorylation under our experimental conditions.

*A. nidulans* PkhA (An-ksg1; AN3110) and Ypk1 (PkcB) orthologs are both essential kinases. The deletion of *pkhA* and *pkcB* results in cells that initiate irregular growth before arresting as microcolonies, consistent with the idea that PkcB is regulated by PkhA [118]. We detected 15 phosphosites in PkhA (AN3110); seven dephosphorylated in H_2_O_2_ and one (S207) H_2_O_2_-specific. Since PkcB essential T-loop phosphorylation was lost in H_2_O_2_, these results suggest that H_2_O_2_ induces a transient inactivation of both PhkA and PkcB. This is consistent with the fact that FpkA, another potential PkcB substrate [161], was dephosphorylated at three phosphosites (S102, S175, and T461), also acquiring two phosphosites (T253 and S256) in H_2_O_2_. Moreover, flipase DnfA, another potential FpkA substrate, was also dephosphorylated at two sites (S78 and T258) and phosphorylated at one (S50) in H_2_O_2_. Both kinases had additional phosphosites, which did not change in the presence of H_2_O_2_ (Figure 2). According to the *S. cerevisiae* paradigm [162], these results suggest that in *A. nidulans*, H_2_O_2_ regulates membrane and cell wall homeostasis through the regulation of TORC2, PkhA, and PkcB.

Although the cAMP-PKA pathway is often related to glucose sensing, the starvation for other essential nutrients such as nitrogen results in the downregulation of PKA targets [163]. This might explain why we also observed phosphorylation changes in response to H_2_O_2_ in cAMP-PKA pathway components (Table 9). The phosphorylation pattern of RasA (AN0182) was not affected by H_2_O_2_. However, it is remarkable that 13 phosphosites were detected in its putative GEF AN2130, the ortholog of *S. cerevisiae* Cdc25; two dephosphorylated (S78, S729) in H_2_O_2_ and seven (S331, S713, S715, S721, S731, S733, T736) H_2_O_2_-specific. Adenylate cyclase CyaA [144] contained eight phosphosites and five were dephosphorylated in H_2_O_2_. The phosphodiesterase PdeA (AN0829) had a single H_2_O_2_-specific phosphosite (S325) and notably, putative kinase AN7572, the ortholog of *S. cerevisiae* Rim15 [118], contained 25 phosphosites; six dephosphorylated and seven phosphorylated in H_2_O_2_.

In summary, TORC1 promotes anabolic processes including protein synthesis, glucose metabolism, and nucleotide and lipid biosynthesis. In *S. cerevisiae*, TOR1C inhibition with rapamycin induces a global pattern of gene expression similar to those induced by nitrogen starvation [164]. As discussed further below, our results are consistent with the inactivation of nitrogen anabolic pathways by H_2_O_2_ and the activation of amino acid catabolism, gluconeogenesis, and the pentose phosphate pathway. These responses are consistent with the inactivation of TORC1, the activation of TORC2, and a downregulation of the cAMP-PKA pathways by H_2_O_2_.

### 3.7. The Phosphorylation of Multiple Proteins Involved in Transcriptional Regulation Is Affected by H_2_O_2_

The phosphorylation of many proteins involved in transcriptional regulation including proteins involved in nuclear-cytoplasmic transport (Table S4) and chromatin-remodeling and transcription factors (Table S5) was affected by H_2_O_2_. Previous work has shown that oxidative stress can affect nuclear transport [165,166,167,168]. The transport of macromolecules in and out of the nucleus is regulated by the nuclear pore complex (NPC), whose overall structure and composition is highly conserved in many organisms [168]. In addition, the NPC regulates chromatin organization, gene regulation, and genome integrity [169]. Using GO terms “protein import into nucleus” (GO:0006606) and “structural constituent of nuclear pore” (GO:0017056), we identified several nucleoporins and karyopherins as phosphoproteins affected by H_2_O_2_. Protein AN3910 had the higher phosphosite number in this category, with 20 total phosphosites; 12 dephosphorylated in H_2_O_2_, one present only in H_2_O_2_, and seven that remained unchanged. AN3910 orthologs in *S. pombe* and *S. cerevisiae* are nuclear inner membrane proteins related to subtelomeric heterochromatin organization. Twelve phosphosites were detected in *A. nidulans* Nup159 (AN2086) with three unique to H_2_O_2_-treated samples. Nup159 is a nuclear pore protein similar to yeast Nup159 and human protein Nup214 [170]. In addition, four phosphosites were identified in nuclear pore complex protein SonB [171], one of which was H_2_O_2_-specific. Interestingly, it has been reported that oxidative stress induces the phosphorylation of human Nup159 and SonB homologs, affecting the exportation of nuclear proteins [165].

In addition to the transcription factors directly regulated by the three MAPK pathways discussed before (Figure 1), H_2_O_2_ induced phosphorylation changes in many other transcription factors (Table S5). Our results confirm phosphosites reported previously and uncover multiple novel phosphosites. For example, the only phosphosite (S421) detected in developmental regulator StuA (AN5836), one (S451) of five phosphosites detected in putative heat shock factor Hsf1 (AN8035), and one (S287) of four present in putative histone acetyltransferase Nmy1 (AN5640), have been reported before as differentially phosphorylated in the absence of MAPK MpkA [36]. Besides novel phosphosites, we detected H_2_O_2_-specific phosphorylation of Hsf1 (T449) and Nmy1 (S304) (Table S5) at motifs that are potential substrates of Cdc2 and PKA kinases, respectively.

We examined the phosphorylation of YapA and SrrA (Figure 1, Table S5), as these transcription factors are directly involved in the antioxidant response in *A. nidulans* and other fungi. In *A. nidulans*, Yap1 homolog NapA is required for the induction of catalase CatB in response to H_2_O_2_ and for resistance to H_2_O_2_ and other types of oxidative stress as well as for the regulation of multiple genes during asexual spore formation [26,27]. Likewise, SrrA is needed for CatB induction and resistance to H_2_O_2_ [28]. H_2_O_2_ sensing by *S. cerevisiae* Yap1 and its homologs involves the oxidation of specific cysteines, which results in its nuclear accumulation and transcriptional activation. However, the phosphorylation of oxidized nuclear Yap1 has also been reported [172], and in *C. albicans,* a correlation between the phosphorylation of oxidized Cap1 and Cap1-dependent gene expression has been reported [173]. Nevertheless, the kinase and target phosphorylation sites on Yap1 and Cap1 are unknown. We detected two phosphosites in NapA; one (S318) unaffected by the presence of H_2_O_2_ and one (T316) H_2_O_2_-specific, predicted as a GSK3 substrate.

SrrA is the homolog of *S. cerevisiae* response regulator Skn7. Like Skn7 [174], SrrA is required to respond to oxidative, osmotic, and cell wall stress [28]. In contrast to Yap1, Skn7 cysteines are dispensable for these functions. As a classical response regulator, its activity in response to osmotic stress depends on the phosphorylation of the conserved aspartate D427 by the Sln1 histidine kinase. However, Skn7 D427 and Sln1 are dispensable for its activation in response to oxidative stress, and instead, it becomes phosphorylated by a different unknown mechanism. Notably, Skn7 oxidant-dependent phosphorylation requires the presence of Yap1, suggesting that a nuclear interaction of Yap1 and Skn7 is necessary for Skn7 phosphorylation, and the corresponding activation of oxidative stress response genes [174]. Although the phosphosite identities and responsible kinases involved in this response are unknown, a role for CDK8 kinase in influencing Skn7 phosphorylation, protein levels, promoter occupancy, and oxidative stress-induced gene expression has been reported recently [175]. In this context, it is interesting that we detected SrrA phosphorylation at T244 in a H_2_O_2_-specific fashion, potentially by kinase Cdk5. The phosphorylation changes of other transcription factors involved in nitrogen metabolism are discussed in Section 3.8.

Other proteins involved in transcriptional regulation found phosphorylated only in the presence of H_2_O_2_ are AN4894, AN2771, AN5048, AN0091, AN5836, and AN3688 (Table S5). AN4894 is the ortholog of SPT7, a subunit of the SAGA complex that plays important roles in the regulation of stress-induced genes in *S. cerevisiae* [176] and in the oxidative stress response in *C. albicans* [177]. Moreover, the SAGA complex participates in the regulation of genes mediated by Hog1 during osmotic stress [178]. We found that a phosphosite detected in AN4894 (S451) only in H_2_O_2_ is conserved in *A. fumigatus* (S456) and *N. crassa* (S477) and is phosphorylated (S439) in *S. pombe* [179]. A single H_2_O_2_-specific phosphosite (S331) was also detected in AN5048. AN5048 is similar to Yox1, which interacts with Mcm1 and ECB proteins to regulate cell cycle in *S. cerevisiae*. Yox1 is also a substrate of the NimX orthologous kinase Cdc28, which we found phosphorylated at two sites, one of them (T14) detected only in H_2_O_2_. AN2771, a putative transcription factor similar to *S. cerevisiae* RBA50 was phosphorylated at S143 only in the presence of H_2_O_2_, possibly by SakA/MpkC, according to NetPhos (Table S5).

Seven proteins identified as putative members of the histone deacetylase complex Rpdl3 showed H_2_O_2_-specific phosphorylation changes. The Rpd3 histone deacetylase complex is required for proper expression of multiple environmental stress genes, under different stress conditions [180] and Hog1 recruits Rpd3 to osmostress responsive genes in *S. cerevisiae* [181]. AN4694, the ortholog of CTI6 was found phosphorylated at T468 and S471 with T468 being H_2_O_2_-specific. Twelve phosphosites were detected in AN1777, five detected only in H_2_O_2_ (Table S5), with S461 possibly phosphorylated by SakA/MpkC (p38), according to NetPhos. These results suggest the participation of the Rpd3 complex in the regulation of genes in response to H_2_O_2_ signaling in *A. nidulans* and a role for SakA/MpkC in this process.

### 3.8. H_2_O_2_ in the Regulation of Nitrogen Assimilation and the Reprogramming of Metabolism

Our results revealed that the phosphorylation patterns of a high number of transcription factors and enzymes involved in nitrogen assimilation were affected by the presence of H_2_O_2_. In filamentous fungi, a preferred nitrogen source such as ammonium or glutamine prevents the expression of genes required for the uptake and metabolism of alternative nitrogen sources, in a process known as nitrogen metabolite repression (NMR, see [182] for a review). The most important regulators of NMR in *A. nidulans*, the transcription factors AreA, AreB, MeaB, TamA, and transcription corepressor NmrA, were detected as phosphoproteins (Table S6). Notably, AreA phosphosites S440, S441, and T749, and TamA phosphosites S175 and S229 were H_2_O_2_-specific, while AreB S183 and NmrA S283 and S289 unique phosphosites were dephosphorylated in H_2_O_2_.

Under our experimental conditions, nitrate has to be transformed to the preferred nitrogen sources of glutamate and glutamine through a pathway that consumes both energy and reducing power. The process involves the transport of nitrate to the cell by transporters NtrA/NtrB, its subsequent conversion to nitrite by nitrate reductase NiaD, and then to ammonia by nitrite reductase NiiA. Ammonia is then transformed to glutamate and glutamine by NADP-glutamate dehydrogenase GdhA and glutamine synthase (GS), respectively. The transcriptional regulation of the nitrate assimilatory pathway is carried out by the nitrate-specific activator NirA and by AreA [183]. As shown in Table S6, H_2_O_2_ induced the phosphorylation of NirA at S725 and S727. Interestingly, in the absence of nitrate, the reversible oxidation of a conserved methionine in NirA nuclear export sequence results in the relocation of inactive NirA to the cytosol [184]. This opens the possibility that NirA might be regulated by two different redox mechanisms involving methione oxidation and H_2_O_2_-mediated phosphorylation. In addition, nitrate transporter NrtB (AN0399) was phosphorylated at T247, while the only two phosphosites detected in putative transporter CrnA (AN1008) were dephosphorylated in H_2_O_2_. H_2_O_2_-specific phosphosites were also detected in nitrogen-related transcription factors AN1927 (S174), AN4489 (S12), and UaY (S922). AN1927 is regulated by nitrate limitation [185] and its ortholog SPCC320.03 increases in response to osmotic and oxidative stress in *S. pombe* [186]. AN4489 is similar to *S. cerevisiae* DAL81, a positive regulator of genes involved in multiple nitrogen degradation pathways [187]. UaY is a conserved transcriptional activator of the purine degradation pathway [188]. We propose that the H_2_O_2_-induced phosphorylation of these transcription factors results in their activation and the consequent induction of amino acid and purine degradation pathways.

The expression of the glutamate dehydrogenase *gdhA* gene is a highly regulated process. It requires AreA [189], a TamA double function (as AreA coactivator and as DNA-binding transcription factor) as well as TamA interaction with the regulator of leucine biosynthesis LeuB [190]. We find it remarkable that AreA, TamA, and LeuB (S151) contained H_2_O_2_-specific phosphosites. It is unknown if such H_2_O_2_-induced phosphorylation results in the activation or inactivation of these transcription factors. However, it should be considered that NADP-glutamate dehydrogenase is itself irreversible, inactivated by ROS [14] and reversibly inactivated by disulfides [12], while glutamine synthetase is also inactivated by ROS [13]. Notably, glutamine synthetase S316 was detected as a single H_2_O_2_-specific phosphosite, and this residue is conserved in fungal and human enzymes. In H_2_O_2_, nitrate reductase NiaD (AN1006) N-terminus was dephosphorylated, while putative nitrite reductase NiiA (AN1007) acquired three phosphosites. A consistent hypothesis is that high H_2_O_2_ levels result in the inactivation of the nitrogen assimilation pathway at both transcriptional and posttranslational levels, perhaps as a transitory mechanism to prevent the futile consumption of energy and reducing power in non-growing conditions, under which amino acid and fatty acid catabolism would be favored. The fact that NAD-glutamate synthase (GOGAT) and catabolic NAD-glutamate dehydrogenase GdhB [191] were dephosphorylated in the presence of H_2_O_2_ (Table S6) suggests that these enzymes might be activated by dephosphorylation. Consistent with this, the reversible inactivation of NAD-GDH by phosphorylation and its activation by dephosphorylation has been reported in *Candida utilis* and *S. cerevisiae* [192,193], although the phosphosites involved in this regulation were not identified. This interpretation is also consistent with the inactivation of nitrogen assimilation enzymes and the increase in proteolysis observed during asexual differentiation in *N. crassa* [15].

### 3.9. The Phosphorylation of Proteosome-Mediated Proteolysis Components Is Affected by H_2_O_2_

Since increased amino acid catabolism is often related to increased protein degradation [194], we analyzed the effects of H_2_O_2_ in the phosphorylation of components related to proteosome-mediated proteolysis including SUMOylation and neddylation (Table S7). The F-box protein SconB, involved in sulfur metabolite repression in *A. nidulans* [195], was dephosphorylated at S303 and phosphorylated at S115 and S281 in the presence of H_2_O_2_. Notably, *S. cerevisiae* SconB ortholog Met30 regulates both cell cycle arrest and methionine biosynthesis during nutritional or cadmium stress. As part of the E3 ubiquitin ligase complex SCF^Met30^, Met30 controls the ubiquitylation of transcriptional activator Met4 and cell cycle inhibitor Met32. Under growing conditions, Met4 is ubiquitinylated by SCF^Met30^ and maintained in a stable transcriptionally inactive state, while Met32 ubiquitylation results in its proteasomal degradation. Under low methionine levels, Met4 is activated by deubiquitylation and by phosphorylation, while Met32 deubiquitylation results in its stabilization and cell cycle arrest [196]. In *A. nidulans*, the Met4 ortholog MetR is also regulated by ubiquitin mediated inactivation or degradation [197]. We propose that H_2_O_2_-induced SconB phosphorylation results in its inactivation and the consequent activation of MetR. MetR is required not only for sulfur metabolism, but also for the expression of multiple genes involved in stress responses and carbohydrate and energy metabolism [197] as well as for the expression of the SakA-related phytochrome FphA and other five histidine kinases [198]. This indicates that MetR is part of a more general stress response that would include cell cycle arrest, and the activation of methionine, cysteine, and glutathione biosynthesis, as part of an antioxidant response.

The phosphorylation of HulE (AN1966) [199], another ubiquitin ligase potentially involved in cell cycle arrest and gene silencing, was drastically affected by H_2_O_2_. HulE contained 14 total phosphosites; six of them dephosphorylated in H_2_O_2_ and three H_2_O_2_-specific. Tom1, a *S. cerevisiae* HulE ortholog, is required for ubiquitination and degradation of F-box protein Dia2 during G1 and G2/M phases of the cell cycle, whereas the Dia2 is stabilized during the S phase [200]. Dia2 is involved in DNA replication and also ubiquitylates Sir4, and cells lacking Dia2 show heterochromatin silencing defects. Recently, it was reported that phosphorylated Tom1 shows a micromolar affinity for Sir4 H-BRCT, leading to the proposal that Tom1 phosphorylation results in Sir4 stabilization and gene silencing [201]. By analogy, HulE H_2_O_2_-induced phosphorylation might regulate cell cycle and gene silencing in *A. nidulans*.

AN3449, a deubiquitinase of the OTU family, was phosphorylated (S97, S98) only in H_2_O_2_. *S. cerevisiae* ortholog Otu1, involved in ER-associated degradation of substrates poly-ubiquitinated by Hrd1, has been detected as phosphorylated at S134 [202]. Although the effect of this phosphorylation is unknown, human deubiquitinase DUBA (OTUD5) provides an example where phosphorylation of a single residue is necessary and sufficient to activate the enzyme [203].

DenA deneddylase, which removes ubiquitin-like protein Nedd8 from target proteins, is required for the normal growth and asexual development of *A. nidulans*. Its stability depends on its phosphorylation status and its localization in either the nucleus or cytoplasm. DenA phosphorylation at S243 and S245 stabilizes the protein during growth and initial asexual development. Later in development, DenA phosphorylation at S253 destabilizes the protein [78]. We detected DenA phosphosites S243 and S253, and a novel site at S241, only in the presence of H_2_O_2_. As indicated before, phosphatase DipA, which controls this phosphorylation-dependent stability of DenA [78], was dephosphorylated at eight phosphosites and phosphorylated on one in H_2_O_2_ (Table S2). This suggests that H_2_O_2_ regulates neddylation levels by the inactivation of DipA and the consequent phosphorylation and destabilization of DenA.

The SumO protease UlpB is required for the production of sexual spores, resistance to DNA damaging compounds, and the production of the secondary metabolite sterigmatocystin [204]. We detected four phosphosites in UlpB, with two of them (S167, S496) being H_2_O_2_-specific. Under the same conditions, SumO protease UlpA single phosphosite S595 was dephosphorylated, suggesting an inverse regulation of these two proteases by H_2_O_2_. On the other hand, a single H_2_O_2_-specific phosphosite (S110) was detected in AN6547, the ortholog of the *S. cerevisiae* Pre5 20S proteasomal subunit. The phosphorylation of different proteosome subunits has been shown to result in increased activity [205].

Two proteins involved in the regulation of carbon source utilization, AcrB (AN3597) and CreD (AN4170), contained H_2_O_2_-specific phosphosites. AcrB, which interacts genetically with the ubiquitination pathway [199], contained two unique phosphosites (S910 and S911) in H_2_O_2_. CreD, containing arrestin domains and PY motifs, is the ortholog of *S. cerevisiae* Rod1 [199]. Rod1 interacts with ubiquitin ligase Rsp5p, contributes to desensitization and internalization of pheromone receptor Ste2, and its function requires calcineurin-dependent dephosphorylation [206]. In H_2_O_2_, we detected that two out of six CreD phosphosites were removed and two were present only in this condition.

### 3.10. H_2_O_2_ in the Regulation of Gluconeogenesis and the Pentose Phosphate Pathway (PPP)

The simultaneous inactivation of nitrogen assimilation and the activation of amino acid catabolism by H_2_O_2_ would result in major metabolic changes including the accumulation of α-ketoglutarate and the induction of gluconeogenesis. α-ketoglutarate has direct (i.e., H_2_O_2_ detoxification) and indirect (i.e., glutathione biosynthesis) antioxidant properties [207], and has been recently involved in chromatin modifications, increased life span, and the maintenance of pluripotency in embryonic stem cells [208]. Gluconeogenesis and the pentose phosphate pathway (PPP) would allow for the generation of NADPH, necessary to maintain the thioredoxin and glutathione systems to contend with high ROS levels. Indeed, a sudden increase in PPP activity is among the fastest known responses to H_2_O_2_ [209]. H_2_O_2_ induced changes in the phosphorylation status of PPP enzymes (Table S8) glucose-6-phosphate isomerase, putative transketolases (AN0688 and AN9180), and putative transaldolase PppA (AN0240). Moreover, glucose-6-phosphate dehydrogenase GsdA (AN2981), a rate limiting enzyme in PPP, contained a unique H_2_O_2_-specific phosphosite (S418) (Table S8). This enzyme is critical for adaptative responses to H_2_O_2_ in *S. cerevisiae* [210] and its activation by phosphorylation is critical for stress resistance in plants [211]. AN1015, a putative phosphorylase that would be involved in glycogen degradation, was phosphorylated at S17 in H_2_O_2_. Overall, these results suggest that H_2_O_2_ induces the PPP through the phosphorylation/activation of specific enzymes in this pathway.

Linking nitrogen and carbon metabolism, the γ-amino butyric acid (GABA) shunt, a conserved metabolic route that bypasses two steps of the Krebs cycle and generates NAD(P)H, has been involved in stress response and ROS mitigation, as GABA accumulates under different stress conditions [212,213,214]. GABA is synthesized from glutamate by glutamate decarboxylase and converted by GABA aminotransferase and succinate semialdehyde dehydrogenase to succinate and NAD(P)H, which are linked to the Krebs cycle and the mitochondrial electron transport chain. In addition, alanine transaminase can utilize alanine to regenerate glutamate and produce pyruvate. We found that in response to H_2_O_2_ *S. cerevisiae* Uga2 succinate semialdehyde dehydrogenase ortholog AN3829 was phosphorylated at T261 and T262, while alanine transaminase Alt1 ortholog AN1923 was phosphorylated on S72. In contrast, putative GABA transporter AN8990 was dephosphorylated at S23 and S531 (Table S8). These results suggest that H_2_O_2_ might modulate the flux of the GABA shunt by regulating the phosphorylation of these proteins.

### 3.11. H_2_O_2_ in the Phosphorylation of Proteins Involved in ROS Metabolism

As shown before, H_2_O_2_ induces phosphorylation changes in multiple regulatory proteins. However, we found that H_2_O_2_ also induced phosphorylation changes in proteins directly involved in ROS metabolism (Table S9), as is the case of catalases CatC and CatB, required for H_2_O_2_ decomposition [24]. CatC showed a single invariable phosphosite (S15), while CatB presented a single phosphosite (S179) only in H_2_O_2_. Interestingly, the activation of CatC by phosphorylation induces increased tolerance to salt and oxidative stress in rice [215]. Peroxiredoxins also participate in the breakdown of H_2_O_2_, but have additional functions in H_2_O_2_ sensing and as chaperones [216]. We detected peroxiredoxins TpxA, TpxB, and PrxA as phosphoproteins. TpxA contained a single invariable phosphosite (S136), TpxB presented a single H_2_O_2_-specific phosphosite (S177), and PrxA unique phosphosite (S30) was dephosphorylated in H_2_O_2_. TpxA and TpxB play minor functions in H_2_O_2_ detoxification and TpxA elimination results in decreased asexual sporulation [27].

On the other hand, NADPH oxidases (NOX) are enzymes that produce ROS in a regulated manner and its regulation by ROS is not known in fungi [9]. *A. nidulans* NoxA and its regulatory subunit NoxR (AN6046) are essential for sexual development and partially required for asexual sporulation [18,217]. NoxR is the homolog of p67phox [218], which is activated by phosphorylation in animal cells [219,220]. We detected three contiguous phosphosites in NoxR (S308, T309, and T310) and one of them (T310) was dephosphorylated in H_2_O_2_. In *N. crassa*, the NOX-1 catalytic subunit is localized in the vacuolar system and the plasma membrane, and NOX-1 and NoxR ortholog NOR-1 colocalize only in discrete vesicular structures [17]. The dephosphorylation of NoxR in response to H_2_O_2_ suggests that ROS produced in one cell compartment (i.e., the cytoplasm or mitochondria) might affect the production of ROS by NOX enzymes in a different cell compartment (i.e., the plasma membrane or vesicular structures).

OXR1 (oxidant resistant) is a protein conserved in eukaryotes and whose depletion from fungi to humans results in increased sensitivity to oxidative stress [221]. Although OXR1 molecular mechanism is not well understood, it is required for the expression of several antioxidant genes [222]. There is evidence in human cells indicating that OXR1 modulates histone methylation and chromatin structure [223]. A role as a ROS sensor protein has also been proposed, based on the presence of a cysteine in the highly conserved C-terminal TLDc domain, which is oxidized by H_2_O_2_ in vitro [224]. The TLDc domain alone is sufficient to confer functionality in mouse [224] and is the OXR1 part that is actually conserved in fungi. *A. nidulans* OXR1 (AN3004, renamed here as OxrA), containing the conserved cysteine, had four phosphosites (S315, T317, T318, and T319) clustered at the C-terminus of the TLDc domain, all of which were dephosphorylated in the presence of H_2_O_2_. S315 and T319 are conserved in the Aspergilli, suggesting that their dephosphorylation in H_2_O_2_ might be important for OxrA function. Three H_2_O_2_-specific phosphosites were detected in AN1100, a hypothetical 5-oxoprolinase potentially involved in glutathione biosynthesis.

## 4. Concluding Remarks

Our results indicate that H_2_O_2_ has profound regulatory effects in cell physiology by affecting signal transduction, nitrogen and carbon metabolism, and gene expression. H_2_O_2_ induced phosphorylation changes in several proteins involved in cell cycle arrest including NimT, Cdc42 regulators, PP2A phosphatase, SconB, HulE, and Aurora kinase, which indicates that this is an important component of the response to H_2_O_2_. We propose that a large part of H_2_O_2_-induced changes in MAPK and TOR signaling, and the crosstalk between these pathways is mediated by its effects on phosphoinositide metabolism. We have provided a set of specific predictions on how ROS affect specific pathways, which can be valuable in designing future experiments.

## Figures and Tables

**Table 1:** H_2_O_2_-induced phosphorylation changes in proteins associated with the stress SakA/MpkC MAPK pathway. Amino acid residues dephosphorylated or phosphorylated in H_2_O_2_ are indicated in blue and red, respectively.

Protein	Phosphosites without H_2_O_2_	Phosphosites with H_2_O_2_
AN9008/FphA	S15, S74, S77, T79, S80	S74, S77, T79, S80, S98, S100, S104, T105
AN3102/PhkA	S94, S95, S452, T479, S480, T481	S89, S90, S92, S94, S95, S96, T479, S480
AN3101/PhkB		S396, T818, S819, S1454
AN2005/YpdA	S3	
AN7697/SskA	T506, T508, S559, S792, S794	T506, S508, S558, S559, S789, S792
AN1180/SskB	S90, T92, T1175	S90, T1173
AN0931/PbsB	S174, S177, S179, S210	S174, S179, S210
AN1017/SakA/Hog1		T171, Y173, T176
AN4668/MpkC	T176	Y173
AN6982/PtpA	S88, S408	S88, S408, S412, S413
AN4483/SrkA	S451	S31, T412, T413, S451
AN4000/FabM	S207	T368, S376
AN2911/AtfA	T159	S132, S136
AN3688/SrrA		T244
AN3469/H2B		S11, T12, S137, S138
AN3941/NimT		S256
AN5744 (Putative 14-3-3 like protein; ortholog of *S. cerevisiae* Bmh2)	S211	
AN4182/NimX/Cdc28	Y15	T14,Y15
AN3648/NimE	S136, S138, S142	S136, S138, S142
AN2412/CmkA		S376
AN8827/CmkC	S34, S475, S553	S34, S140, S475

**Table 2:** H_2_O_2_-induced phosphorylation changes in proteins associated with the pheromone MpkB MAPK pathway. Amino acid residues dephosphorylated or phosphorylated in H_2_O_2_ are indicated in blue and red, respectively.

Protein	Phosphosites without H_2_O_2_	Phosphosites with H_2_O_2_
AN2269/SteC	S197, S389, S535	S197, S389, S535
AN7252/SteD	S112, S243, S311, Y319	
AN2701/HamE	S425, S901, S966, S973, S1037, S1039, S1040, S1043, T1111, S1112, S1115, S1503, S1536, T1538	S425, S973, T1001, S1004, T1005, S1037, S1039, S1040, T1111, S1112, S1115,
AN3422/Ste7/MkkB	S359, S383	S383
AN3719/MpkB	Y184	T182, Y184
AN6190/SipA	S7, S16, S334, S335	S334, S335, S337
AN6611/SipC	S85, T415	T84, S85
AN4632/SipD	S482	S482
AN4085/SipF	T614, S615	S615
AN8071/StrA	S248, S3798	S248, S379
AN6190/SipA	S7, S16, S334, S335	S334, S335, S337
AN6611/SipC	S85, T415	T84, S85
AN4632/SipD	S482	S482
AN4085/SipF	T614, S615	S615
AN0807/LaeA	S39, S70	S39, S70
AN1052/VeA	S243	S374
AN2290/SteA	S550, T552	S550, T552
AN1959/VosA		T247
AN6243/ImeB	T211, Y212, S214, T215	T45, S48, T55, T210, T211
AN3090/GanA	S288	

**Table 3:** H_2_O_2_-induced phosphorylation changes in proteins associated with the cell wall integrity MpkA pathway. Amino acid residues dephosphorylated or phosphorylated in H_2_O_2_ are indicated in blue and red, respectively.

Protein	Phosphosites without H_2_O_2_	Phosphosites with H_2_O_2_
AN4674 (Membrane protein Wsc4, putative; similar to of *S. cerevisiae* Wsc4	T267, S283	S283, S286
AN4719/Similar to Rom1/2 Rho guanine nucleotide exchange factor; similar to of *S. cerevisiae* Rom2	S277, S280, S281, S796, S797	T42, T195, S277, S280, T281, S796, S797
AN7576 (Predicted Rho GTPase activating protein, similar to *S. cerevisiae* Lrg1)	S83, S655, S679, S680, S764, S1163, T1164, S1165, T1166, S1168, S1170	S83, T92, T93 S655, S679, S680, S764, S1163, T1164, S1165, T1166, S1168
AN11994 (Predicted Rho GTPase activating protein, similar to of *S. cerevisiae* Bag7)	S306, S313, S315, S316, S318, S335, S337, T340, S341, S344, S348, T352, T366, S372, S394, S474, S627, T729, S731, S740, S741, T824	S313, S315, S316, S318, S323, S335, S337, S364, T366, S372, S378, T389, T729, S735, S740, S741,
AN0106/PkcA	S586, S588, S594	S586, S588, S590, S591, S594
AN3110/PkhA (ortholog of *S. cerevisiae* Pkh2)	T134, S136, S150, S201, S203, S424, S425, T429, S617, S619, S755, S756, S759, S760, T764	T134, S136, T207, S424, S425, S759, S760, T764
AN4887/BckA	S763, T767, S955, S979, S983, S1003, S1014, S1033, T1037, S1059, S1061, T1067, S1234	S719, S763, S979, S983, S1003, S1014, S1234
AN4189/MkkA	S117, S121, T144, S149, S169, T367	
AN5666/MpkA	T100, Y102, T105	Y98, T100, Y102
AN2984/RlmA	S317, S337	T315, S317, S337

**Table 4:** H_2_O_2_-induced phosphorylation changes in proteins associated with the Rho GTPase Cdc42 module. Amino acid residues dephosphorylated or phosphorylated in H_2_O_2_ are indicated in blue and red, respectively.

Protein	Phosphosites without H_2_O_2_	Phosphosites with H_2_O_2_
AN7698/ShoA	S138, T207, S215	S138
AN7041/MsbA	S788	
AN5592 (Predicted guanine nucleotide exchange factor ortholog of *S. cerevisiae* Cdc24)	S565, T579, S582, S627, S636, S666, S713, S715, S716, S760, S764, T766, T799, S800	S525, S565, T579, S582, S584, S627, S636, S666, S670, S713, S715, S716, S760, S764
AN0113/Bud3	S229, T232, T480, S792, S818, S838, T1071, S1250, T1251, S1252, S1257, S1269	S229, T232, T794, S818, T820, S838, T840, S1108, S1164, T1251, S1252, S1302
AN3746 (Rho GTPase activator Bem2, putative)	S50, S160, S161, S236, S238	S160, S161
AN7030/BemA	T108, S115, S467, S470, S471, S476, S527, S528, T530, S533, T534	S467, S476, S478, S527, S528, T530, S533, T534
AN2067/Ste20	S44	S44
AN8836/Cla4	S295, S309	T64, T66, S295, S309
AN5787 (GTPase activator activity, role in negative regulation of Rho protein signal transduction, similar to of *S. cerevisiae* Bem3)		S475, S616

**Table 5:** H_2_O_2_-induced phosphorylation changes in proteins involved in phosphoinositide metabolism (kinases, phosphatases, and phospholipases). Amino acid residues dephosphorylated or phosphorylated in H_2_O_2_ are indicated in blue and red, respectively.

Protein	Phosphosites without H_2_O_2_	Phosphosites with H_2_O_2_
Phosphatidylinositol kinases		
AN2766/MssA (Putative phosphatidylinositol-4-phosphate 5-kinase; similar to of *S. cerevisiae* Mss4)	S65, S68, S90, S191, S196, S198, S208, S209, S258, S262, S270, S271	S68, T181, S182, S196, S198, T200, S208, S209, S246, S247, S258, S262, T264, S271, T279, S284
AN4278/Stt4/SstA	Y788, S790, T792	
AN10791/Lsb6	T64, T65, S84, S467, T671, S672, S675	T64, T65, S84, S467, T671, S672, S675
AN5797/VlpA (inositol polyphosphate kinase)	S94, S672	S672
Phosphatidylinositol transfer protein		
AN3709 (CRAL/TRIO domain protein, similar to *S. cerevisiae CSR1)*	T168, S174	
Phosphatidylinositol kinase regulator		
AN5857/Vac7	T422, S424, S350, Y463, T464, S465, S466, S575, S589	S292, S350, S455, T459, T462, Y463, T464, S465, S466, T570, S575, S589, S795
phosphatidylinositol phosphatases		
AN7745 (Similar to *S. cerevisiae* INP52)	S17, S957	S957
AN8053/INP52	S346, S350, S359	
AN0925 (Phosphoric ester hydrolase, similar to *S. cerevisiae* Sac1)	S257, S258, S259	S255, S257, S258, S259
Phospholipases		
AN10413 (Phosphatidylinositol-3-phosphate binding, phospholipase D activity)	S135, S167, S189, S204, T205, S208, S224, S294, S544, Y555, S581, S582, S590, S591, T593, T599, S1336	S39, S47, S135, S189, S204, T205, S208, S294, S581, S582, T586, S590, S591, T593, T599, T603, S1336, S1338, S1461
AN2947/PlcA (Putative 1-phosphatidylinositol-4,5-bisphosphate phosphodiesterase, phospholipase C)	S258, S262	S258, S262, S263
Known effectors		
AN5705 (Ortholog of *S. cerevisiae* Vps74)	S29, S32, S36	S25, S29, S32
AN2496 (Similar to *S. cerevisiae* Efr3)	T254, T794, S795, S850, S854, S924, S1053, S1146, T1148, Y244, S245,	S245, T248, T254, T794, S795, S850, S854, S924, S1053, S1146, T1148
AN0127 (Phosphatidylinositol-3,5-bisphosphate binding, phosphatidylinositol-3-phosphate binding, phosphatidylinositol-4-phosphate binding, ubiquitin binding activity, ortholog of *S. cerevisiae* ATG18)	S283, S285, S286, T296, S322	S283, S286, S288, T296, T321, S322
AN0576/Vps15	S209	S209
AN6351/ATG20	T255, S256, S260, S261, S504	S260, S261, T503, S504, S505
AN4416/PepA	T210, S148, T149, S195	S148, T149, S195
AN2224/SogB	S523, S525, S527, S547	S523, S525, S527, S547
AN4666 (Phosphatidylinositol-3,5-bisphosphate binding, ortholog of *S. cerevisiae* Hsv2)	S270	S270
AN11104 (Protein with a predicted role in actin assembly; similar to *S. cerevisiae* Las17)	S164, T315, S321, S355	S280

**Table 6:** H_2_O_2_-induced phosphorylation changes in FYVE domain containing proteins. Amino acid residues dephosphorylated or phosphorylated in H_2_O_2_ are indicated in blue and red, respectively.

Protein	Phosphosites without H_2_O_2_	Phosphosites with H_2_O_2_
AN1060/kdmA	T246, S249	
AN0986 (PHD finger domain protein	S73, S87, S358, S424	S358, S424
AN2857/Pho23	S349, S467	S349
AN3668 (PHD finger domain protein, putative)	S509, S510, S707, S708, S711, S755, S756	S509, S510, S755, S756
AN4694 (Rpd3L complex)	S471	T468, S471
AN5640/Nmy1	T285, S287, S313	T285, S287, S304, S313
AN6675/NTO1	S11	S6, S11
AN7300 (Similar to *S. cerevisiae* Rco1)	S260, T439, S441, T443	
AN8211	S25, T298, S1297, S1301, S1520, T1623, S1640	T298, S402, S1297, S1301, S1520, T1623, S1640
AN8939 (PHD finger protein)	S199	
Cti6/AN1777	T48, S52, S180, T182, T448, S458, S463	S180, S217, T448, T450, S453, S458, T460, S461
AN0044	S143, S396	
AN7422 (Ubiquitin carboxyl-terminal hydrolase)	S350	S350
AN4637	S60	
AN5167 (Phospholipid binding, zinc ion binding activity)	S346, S349, S407, S410	S349, S617, S620, S621
AN5891 (PHD finger and SET domain protein, putative)	S186, S188	
AN6136	T401	
AN9421 (RING finger domain protein, putative)		S199
AN4497 (MIZ zinc finger domain protein)	S133	S133
AN5516 (Meiosis specific protein Hop1, putative)	S398, T302, S304	
AN10822/SizA	S36, S304	

**Table 7:** H_2_O_2_-induced phosphorylation changes in PH domain containing proteins. Amino acid residues dephosphorylated or phosphorylated in H_2_O_2_ are indicated in blue and red, respectively.

Protein	Phosphosites without H_2_O_2_	Phosphosites with H_2_O_2_
AN7037/Vps36	S269	
AN7750/PSY2 Subunit of protein phosphatase PP4 complex	S803, S804, T805, S806, S807, T809, S820, S822	S803, S804, T805, S806, S807, S808, T809, S822
AN7783/ROM2 similar	S520, T522, S525, S733, S735	S520, T522, S525, S636, Y639, S735
AN0560/EXO84	T157, S158, T159, S418	T157, S158, T159
AN0084/YRB1	T54, S55, S57, S97	S97
AN5102/SPT16	S587	
AN5485/Nup2	T762, S764, S895, S1005, T1269, S1270	T1269, S1270
AN3674/Meu6	S173, T155, S161, T289, S290, S372, S373, S442, T457, S458, S462, S470, S493	S173, S372, S373, S493, T457, S458, S462, S470
AN4601/ATG26	S125, T461, S485, S490, S507, S509, T510, S596, T597, T598	S490, S509, T510
AN6304/Sin1	T73, T75, S337, S338, S342	S337, S338, S342
AN3438/SEC7	S114, T134, S136, S166, S208, S211, S213, S214, S237, S487, S490, S491, S910, T912, S1033, S1035, S1348	S164, S166, S207, S208, S211, S237, S283, S487, S490, S491, S910, S1035, S1348, S1349
AN3424/OSH3	S49, T54, S68, S180, S391, S402	S180, S391, S402
AN2749/SKG3	S16, S17, S43, S478, S468, S718	S468
AN5829/NUM1	S230, S234, S657, S892, S935, S1167, S1177, S1178, S1179	S378, T656, S657, T799, S806, S833, S935, S1179

**Table 8:** H_2_O_2_-induced phosphorylation changes in PX domain containing proteins. Amino acid residues dephosphorylated or phosphorylated in H_2_O_2_ are indicated in blue and red, respectively.

Protein	Phosphosites without H_2_O_2_	Phosphosites with H_2_O_2_
AN6351/ATG20 Ortholog(s) have phosphatidylinositol-3-phosphate binding activity and role in autophagy of mitochondrion	T255, S256, S260, S261, S504	S260, S261, T503, S504, S505
AN3584/SNX4 Ortholog(s) have phosphatidylinositol-3-phosphate binding activity	S17	
AN7030/BemA	T108, S115, S467, S470, S471, S476, S527, S528, T530, S533, T534	S467, S476, S478, S527, S528, T530, S533, T534
AN10918/MVP1 calcium ion binding, phosphatidylinositol binding activity	S236, S247, S271, S274, T276	S236, S247, S271, S274, T276, S353
AN2224/SogB Endosomal phosphatidylinositol-3-phosphate binding retromer complex subunit	S523, S525, S527, S547	S523, S525, S527
AN5787/Bem3		S475, S616
AN4551/AN11912 (Phosphatidylinositol binding, SNAP receptor activity)	S15	S15
AN10350 phosphatidylinositol binding activity and role in cell communication	S298, S302, S817, S818	S298, S302, S322, S817, S818
AN6351/ATG20 Ortholog(s) have phosphatidylinositol-3-phosphate binding activity and role in autophagy of mitochondrion	T255, S256, S260, S261, S504	S260, S261, T503, S504, S505

**Table 9:** H_2_O_2_-induced phosphorylation changes in proteins involved in TOR and cAMP-PKA signaling. Amino acid residues dephosphorylated or phosphorylated in H_2_O_2_ are indicated in blue and red, respectively.

Protein	Phosphosites without H_2_O_2_	Phosphosites with H_2_O_2_
AN5982/TorA	S1905, S2285, S2288, S2308	S1905
AN6304/Sin1/Avo1	T73, T75, S337, S338, S342	S337, S338, S342
AN4639/RAPTOR/KOG1	T13, S14, S19, S20, S51, S960, S987, S990	T13, S14, S19, S51, S960, S985, S987,
AN10756/AN5958/TSC11/Rictor/Avo3	S104, S105	S104, S105, Y108
AN1335/LST8	S267	S273
AN7681/Maf1	S82, S83, T147, T151, S154, S156, S233, T235, S237, T242, T255, S259, S261, T260, S305	S82, S83, T147, T151, S156, T255, S259, S261, S305
AN5973/PkcB/Gad8	T450, S633	S633
AN3110/Ksg1/Pkh1/2	T134, S136, S201, S203, S424, S425, T429, S617, S619, S755, S756, S759, S760, T764	T134, S136, S207, S424, S425, S759, S760, T764
AN7750/Psy2	S716, S803, S804, T805, S806, S807, T809, S820, S822	S803, S804, T805, S806, S807, S808, T809, S822
AN4238/SchA	S240, S769	S227, S233, S240, S359, S360, S769
AN4171/Slm2	S31, S33, T472, T481, T482, S494	S31, S33, S468, T472
AN5671/Slm1	S75, S79, S135, S612, S656	T73, S75, S132, S135
AN0802/PAH1	S112, S117, T118, S147, T188, S190, S222, S224	S112, S182, S224
AN6590/TSC2	S459, S462	S459, S779
AN4987/PkaR	T37, S38, T119, S120	T37, S38, S53
AN4880/Psk1/YPK3	S46, S389	
AN7537/Ppk33/YPK2	S403, T404, S406, S414, T415, S418, S541	S414, S418, S541
AN1933/ORM1	S6	S6
AN2464/LagA/LAG1	S7, S8, S12, S30, S387, T401, S403, T404	S8, S12, S30, S387, S400
AN1545/PabA/CDC55	S154	S417, S419, T421, S422, S423
AN9467/ParA/RTS1	S585, T586, T587	S147, S585, T586, T587
AN4085/TPD3	T614, S615	S615
AN0144/FpkA/Nrc2/FPK1	S102, S175, S255, S258, S437, T455, S457, T461	T253, S255, S256, S258, S437, T455, S457
AN8672/DnfA/DNF1	S78, S79, S255, T258, T259, S261, S316, S320, S323	S50, S79, S255, T259, S261, S316, S320, S323
AN0351/GfdA/GPD1	S272, S277, S279, S281, T282	S279
AN1343/NEM1	S27, S153, S157, S309, S383	S153, S158, S309
AN10545/WHI2	S103, S106, S107, S111	S107, S109, S111
AN5815/Aurora	S80	
AN0182/RasA	S177, S180, S182	S177, S180, S182
AN2130/Cdc25	S55, S68, S78, S80, S729, S749	S55, S68, S80, S331, S713, S715, S721, S731, S733, T736, S749
AN3913/CyaA	S121, S143, S399, S400, S401, S405, S1143, S1153	S400, S401, S405
AN0829/PdeA		S325
AN7572/SrrB/Rim15	S353, T592, S593, S641, S648, S649, S650, S665, S683, S687, S977, S979, S982, S1433, S1437, T1471, S1474, S1819	T34, S38, S353, S641, S648, S649, S650, S665, S979, S982, S1433, S1436, S1437, S1448, T1467, S1469, T1471, S1474, S1931

## Data Availability

The mass spectrometry proteomics data are available as Supplementary Materials.

## Supplementary Materials

The following are available online at https://www.mdpi.com/article/10.3390/jof7080624/s1, Supplementary File S1: Phosphopeptide and protein database; Table S1. H_2_O_2_-induced phosphorylation changes in putative RNA-binding proteins, Table S2. H_2_O_2_-induced phosphorylation changes in putative phosphatases, Table S3. H_2_O_2_-induced phosphorylation changes in proteins involved in secondary metabolism, Table S4. H_2_O_2_-induced phosphorylation changes in proteins involved in nucleo-cytoplasmic transport, Table S5. H_2_O_2_-induced phosphorylation changes in proteins involved in chromatin remodeling and transcription factors, Table S6. H_2_O_2_-induced phosphorylation changes in proteins involved in nitrogen metabolism, Table S7. H_2_O_2_-induced phosphorylation changes in proteins involved in proteosome-mediated proteolys, Table S8. H_2_O_2_-induced phosphorylation changes in proteins involved in gluconeogenesis and the pentose phosphate pathway, Table S9. H_2_O_2_-induced phosphorylation changes in proteins directly involved in ROS metabolism.
