# Supplementary material for: H2O2 Induces Major Phosphorylation Changes in Critical Regulators of Signal Transduction, Gene Expression, Metabolism and Developmental Networks in Aspergillus nidulans
Source: J Fungi (Basel). 2021 Jul 31;7(8):624. doi: 10.3390/jof7080624 (PMC8399174; doi:10.3390/jof7080624)
Supplement: Supplementary file 1 [file jof-07-00624-s001.zip › Supplementary Tables S1-S9.pdf]

Table S1. H_2O_2-induced phosphorylation changes in putative RNA-binding proteins

Protein	Phosphosites without H_2O_2	Phosphosites with H_2O_2
AN1325/Pbp1	T367, S369, S429, S633, S634, T640	S267, S429, S634
AN7474/JSN1	S67, T140, S143, S207, T239, S251, S253, T401, S556, S557, S570, T572, S971	S67, S69, S207, S251, S253, S331, S333, T401, S557, T561, S570, T572, S971
AN5802/SwoK	T84, S121	T84, T103, S121
AN10257/Pbp2	S19, S20	S19, S20
AN8293/Utp21	S865	
AN2068/Vgl1	S187, S832, T836, S838, S842	S187, S832, T836, S838, S842
AN4748/Nop6	S57	S57
AN7700/Whi3	S280, S615	T216
AN8276	S430, S440	S402, S423, S440, S460
AN3072/Nsr1	S112	S112, S674
AN7706/Psb1	Y74, S77	S75, S81
AN6695/NmdA	S993, S995	

Table S2. H_2O_2-induced phosphorylation changes in putative phosphatases

Proteins	Phosphosites without H_2O_2	Phosphosites with H_2O_2
An5057/CdcA	S61, T415, S419, T430, S484, T482, S484, S602	S61, S63, T415, S419, T430, S484, S605
AN10077/AN0425/PsrA	S93, T95, S96, S207, S251, S276	S151
AN10593 (Has domain(s) with predicted phosphatase activity; similar *to S. cerevisiae* Ser2)	S21, S22, S55	S21, S22
AN10563/Pho8	S17, S18, S150	S14, S17
AN1358 (Ortholog(s) have protein serine/threonine phosphatase activity; similar to *S. cerevisiae* Ptc2)	T390	T390
AN0914/PtcD	S427	S31, S32, T33, T34, S427
AN4967 (Acid phosphatase, putative; Ortholog of *S. cerevisiae* Pby1)	S507, T534, T535	T534, T535
AN4057 (Has domain(s) with predicted protein tyrosine/serine/threonine phosphatase activity; ortholog of *A. fumigatus* DspB)	S101	
AN0129/PpsA	S46, S49, S50	
AN4713 (Ser/Thr protein phosphatase family; similar to *S. cerevisiae* YNL217W)	S20	
AN10946/DipA	T479, S480, S488, T490, S494, T495, T496, S505, T511, S517, T518, S526	T479, S480, S488, T490, S536

AN7750/Psy2	S716, S803, S804, T805, S806, S807, T809, S820, S822	S803, S804, T805, S806, S807, S808, T809, S822
AN4896/PtpB	S32	
AN6892/PtcA	S6, S7, S41, S74, S86, S87, S97, S498, T500	S86, S87, S97, S498

Table S3. H_2O_2-induced phosphorylation changes in proteins involved in secondary metabolism

Protein	Phosphosites without H_2O_2	Phosphosites with H_2O_2
AN1034/AfoE	S2384, T2385	
AN3612/CeM1		S874, T875, T877
AN2547/EasB	S1730, S1731	
AN9407/FasA	S559, S560	S162, S-559, S-560
AN9408/FasB	S5, T6, T10, T14, T2083, T466	S5, T6, T14, S1147
AN7873/Fas1		T533, T535, T546, S554, S554, T555
AN0640/BasA	S363, S371, S376, S377, S378, T408, S410	S363, S371, S376, T408, S410, S412,
AN0981/Elo1	S470, S-533	S470, S533, T474
AN4592/Sld1	S115, S138, T140	
AN7815/StcJ		S88, T90
AN3380/PkiB	S1564	

Table S4. H_2O_2-induced phosphorylation changes in proteins involved in nucleo-cytoplasmic transport

Proteins	Phosphosites without H_2O_2	Phosphosites with H_2O_2
An5627/SonB	S536, S571, S572	S536, S569, S571, S572
An2086/Nup159	T573, S795, S766, S851, S856, S997, S999, S1035, S803	S795, S803, S851, S1035, S571, S572, T573, S782
AN9109/Nup85	S56, S202, T232, S997, S1029, S1030, S1031, S1033	S56, S202, S997, T1027, S1029, S1030, S1031, S1033
An1190/Nup84	S13, S843, S844, S846, S858, S859, T861, S862	S844, S846, S858, S859, T861
An4293/Nup133	T21, S37, T1271, S1274, S23, S26	T21, S23, S26, S37, S40
An5485/Nup2	T762, S764, S895, S1005, T1269, S1270	T1269, S1270
An4417/Ndc1	S415, S439	S415, S439, S463
An6980/Nic96	S163, T296, S179	S142, S287, T296
An1238/Nup120	S1234	S1234
An6143/Nup82	T824, S825, S821, T822	T817, S821, T822, T824, S825
AN6125/NudE	S532	T469
AN0753 (Nuclear segregation protein (Bfr1), putative; similar to *S. cerevisiae* Brf1)	S373	S373
AN4581 (RNA binding activity and role in RNA metabolic process; similar to *S. cerevisiae* Sgd1)	S562	
An1157/Gle1	S12	S12
An5499/Mlp1	T71	

An1188 (U2 snRNP, U2type spliceosomal complex localization; similar to *S. cerevisiae* Cus1)	S15	
An5627/SonB	S536, S571, S572	S536, S569, S571, S572
An2086/Nup159	T573, S795, S766, S851, S856, S997, S999, S1035, S803	S795, S803, S851, S1035, S571, S572, T573, S782
AN9109/Nup85	S56, S202, T232, S997, S1029, S1030, S1031, S1033	S56, S202, S997, T1027, S1029, S1030, S1031, S1033
An1190/Nup84	S13, S843, S844, S846, S858, S859, T861, S862	S844, S846, S858, S859, T861
An4293/Nup133	T21, S37, T1271, S1274, S23, S26	T21, S23, S26, S37, S40
An5485/Nup2	T762, S764, S895, S1005, T1269, S1270	T1269, S1270
An4417/Ndc1	S415, S439	S415, S439, S463
An6980/Nic96	S163, T296, S179	S142, S287, T296
An1238/Nup120	S1234	S1234
An6143/Nup82	T824, S825, S821, T822	T817, S821, T822, T824, S825
AN6125/NudE	S532	T469
AN0753 (Nuclear segregation protein (Bfr1), putative; similar to *S. cerevisiae* Brf1)	S373	S373
AN4581 (RNA binding activity and role in RNA metabolic process; similar to *S. cerevisiae* Sgd1	S562	
An1157/Gle1	S12	S12
An5499/Mlp1	T71	
An1188 (U2 snRNP, U2type spliceosomal complex localization; similar to *S. cerevisiae* Cus1)	S15	
AN3910 (Establishment of mitotic sister chromatid cohesion, maintenance of rDNA; Ortholog to *S. cerevisiae* Src1)	T67, S68, T95, S97, S98, S100, S101, S115, T116, T175, S206, S235, T239, S249, T251, S665, S674, T669, S674	T67, S68, T95, S97, S98, T175, S219, S665
An0084 (Ran GTPase binding activity; Ortholog to *S. cerevisiae* YRB1)	T54, S55, S57, S97	S97
AN6006/KapD	S70	S70, T71
AN2120/KapJ	S705	S705
An0906/KapB	S872	S872
AN8787/KapM	T430	T430
AN6734/KapF	T920, S922	T920, S922
An7182/Brr6	S125	S440
AN7659 (RNA helicase activity, RNAdependent ATPase activity, inositol hexakisphosphate binding activity; Ortholog to *S. cerevisiae* DBP5)	S41	
AN6374 (DNA helicase activity, DNA/RNA helicase activity, RNA helicase activity; similar to *S. cerevisiae* DBP9)	S358	S358

Table S5. H_2O_2-induced phosphorylation changes in proteins involved in chromatin remodeling and transcription factors

Protein	Phosphosites without H_2O_2	Phosphosites with H_2O_2
AN4502 (bZIP transcription factor, putative)	S54, S58	
AN9404 (C2H2 finger domain protein, putative)	S162, T164	
AN4418 (C2H2 finger domain protein, putative)	S241, T334	S241, T334
AN12489/AN9538 (DNA binding, nucleic acid binding, zinc ion binding activity, role in transcription)	S262	S262
AN10059 (C6 finger domain protein, putative)	S321	S321
AN4773 (C6 transcription factor, putative)	S2, S3, T4	
AN3684 (C6 transcription factor, putative)	S166	
AN4034/HapC	S146, S147	
AN4878 (CP2 transcription factor, putative)	S8	
AN9492/AmdX	S125, T126, S146, S151, S480, S496, S498, S499	S125, T126, S496, S497, S498, S499
AN7050/FarA	S719, T723, S743	T723
AN0937 (DNA binding, DNA binding transcription factor activity)		S647
AN10548/AN4350 (Putative Zn(II)2Cys6 transcription factor)	Y211, T212, T213, S220	
AN3435/LreA	S363, T366	S363, T366
AN1927 (Putative Zn(II)2Cys6 transcription factor)		S174
AN2136 (Putative Zn(II)2Cys6 transcription factor)	T580, S581, S582, Y587	S187, S284, S292, S579, T580, S581, S582
AN2852 (Putative Zn(II)2Cys6 transcription factor)	T744, S761	
AN4489 (Putative Zn(II)2Cys6 transcription factor)	S9	S9, S12
AN6091 (Putative Zn(II)2Cys6 transcription factor)	S161	S161
AN7508 (Putative Zn(II)2Cys6 transcription factor)	S283, S375, S377, S748	S283
AN8885 (Putative Zn(II)2Cys6 transcription factor)	S137	
AN7170/SrbB	S131, S142	S131, S142
AN2854 (Putative forkhead transcription factor)	S20, S310	S20, S143, S310
AN4985 (Putative forkhead transcription factor)	S 651	

AN8858/McnB	S54, T55, S56, S634	
AN1729/PrnA	S72	S72
AN2597 (Putative Zn(II)2Cys6 transcription factor)	S61, S68	S61, S68, T70
AN3050 (Putative Zn(II)2Cys6 transcription facto)	S206	S206
AN3683 (Putative Zn(II)2Cys6 transcription factor)	T673, S674, S675, S677, S679, T680	
AN4558 (Putative Zn(II)2Cys6 transcription factor)	S158, S167, T169, S186	S156, S158, S167
AN5849 (Putative Zn(II)2Cys6 transcription factor)	S554, T557, T637, S653, S946	S554, T557, S946
AN5924 (Putative Zn(II)2Cys6 transcription factor)	S206	S206, S211
AN6889 (Putative Zn(II)2Cys6 transcription factor)	S83, S301	S83, T294, S295, S301
AN7332 (Putative Zn(II)2Cys6 transcription factor)	S51, S53, S55, S56	
AN8161 (Putative Zn(II)2Cys6 transcription factor)	S85, S86, S92	S86
AN8694 (DNA binding transcription factor activity)	S322	S322, S328
AN9147 (Putative Zn(II)2Cys6 transcription factor)	S231	S231
AN1777/Cti6 (Rpd3L)	T48, S52, S180, T182, T448, S458, S463	S180, S217, T448, T450, S453, S458, T460, S461
AN2857/Pho23 (Rpd3L)	S349, S467	S349
AN3668 (PHD finger domain protein, putative)	S509, S510, S707, S708, S711, S755, S756	S509, S510, S755, S756
AN7300 (PHD finger domain protein, putative)	S260, T439, S441, T443	
AN0986 (PHD finger domain protein)	S73, S87, S358, S424	S358, S424
AN6675 (PHD finger domain protein, putative)	S11	S6, S11
AN8939 (PHD finger protein)	S199	
AN8676/McmA	S39	S13
AN2911/AtfA	T159	S132, S136
AN7513/NapA	S318	T316, S318
AN0134/RmtC	T529	
AN4493/ RpdA	S447, S449, S496	S447, S449, S496
AN1944 (Zinc knuckle domain protein)	S6	
AN4894 (Transcriptional activator spt7)		S451
AN4694 (Transcriptional regulator (Cti6), putative)	S471	T468, S471
AN9538/AN12489 (DNA binding, nucleic acid binding)	S262	S262
AN8211 (PHD transcription factor (Rum1), putative)	S25, T298, S1297, S1301, S1520, T1623, S1640	T298, S402, S1297, S1301, S1520, T1623, S1640

AN0162 (Putative APSES transcription factor)	S170, S172, S173	S170, S172, S173
AN0153 (Putative Myb like transcription factor)	S54	S52, S54
AN1402 (Putative Zn(II)2Cys6 transcription factor)	S46, S92, T93, S94, S96, S109, S111, S138, T143, S150, T154, S176, S177, T178, T274, S345, T372, S373, S390	S46, S92, T93, S94, S109, S126, S138, S345, S373, T388, S390,
AN2771 (Transcription factor Rba50, putative)		S143
AN6696 (Chromatin binding activity, role in cellular response to DNA damage stimulus)	S83, S240, S243, S276	S240, S243, S276,
AN0766/PacX	S17, S19, S256	S19, S256
AN4585 (CCR4 NOT transcription complex, subunit 3)	T363, S365, T392, S396, S255, S280, S390, T392	S255, S280, T392, S396
AN6195/CreA	S193	S193
AN1944 (Zinc knuckle domain protein)	S6	
AN8035 (Hsf1)	T91, S302, S451, S485	T449, S451, S485
AN2278 (Catalytic subunit of the SWI/SNF chromatin remodeling complex)	S500, S502	
AN6705/RSC8	S247, T249	
AN1440/AN10191 (AAA family ATPase, putative)	S160, S1496	S160, S1496
AN0689/FacB	S655, T653	T653
AN8687 (Role in regulation of transcription)		S165
AN6505/RcoA	T190	T190
AN3120/PrtT	S20, T154	S5
AN6715 (Putative APSES transcription factor similar to MbpA)	S230	
AN5048 (Homeobox transcription factor, putative)		S331
AN0091/Dot1		S56
AN8825/Set2	S46, T552	
AN5795/Set1	S703	S703
AN6147/Set9	S461, T467	
AN1060/KdmA	T246, S249	
AN10956/Esa1	S113, S117, T119, S171	S113, S117, T119, S171
AN5640/Nmy1	T285, S287, S313	T285, S287, S304, S313
AN3071/Nmy2	T302	
AN2487 (Chromatin remodeling complex subunit (Arp5), putative)	T567, S571, S722	T567, S571, S722
AN1286/Hir1	S1059	S1059
AN1453/Dep1	S102, S104	
AN1375/Rtx2	S26	S26
AN3178/Sds3	S29, S31, T34, S263	S29, T34, S363
AN1546/Sin3	T356, S629, T631	T631
AN2421/FlbC	S150, T186	S150, T186
AN5836/StuA		S421

AN1937/WetA	T142	
AN6578/OsaA	S131, S125, T382, S386, T392, S421	S131, S133, S361, T385, S386, S391, S399, S413, S421
AN0807/LaeA	S70	S70
AN5893/FlbA	S98, S409, S410, S412, S414, S416, S418, S702	S98, S409, S410, S413, S414, S416, S418, S420, S702

Table S6. H_2O_2-induced phosphorylation changes in proteins involved in nitrogen metabolism

Protein	Phosphosites without H_2O_2	Phosphosites with H_2O_2
An8667/AreA	S 234, S 619, T 620, S 744, S 766, S797	S440, S441, S619, S744, T749, S766
AN2944/TamA	S160	S160, S175, S229
AN44867/LeuB		S 150
AN6221/AreB	S183	
NmrA	S283, S89	
AN0098/NirA		S725, S727
AN1927		S174
AN4489	S9	S12
AN0891/UaY		S922
An1006/NiaD	S2, T3, T4, T10, S12, T16, S605, S606,	S605, S606
AN1007/NiiA	S404, S407	S404, S407, T643, T1095, S1097
AN7367 (Activity and role in nitrogen compound metabolic process (nitrilase); similar to *S. cerevisiae* NIT1)	S343	S2, S11, T13
AN1008/CrnA	T277, T279	
AN0439 (Nitrogen permease regulator; role in cellular response to nitrogen starvation, negative regulation of TORC1; similar to *S. cerevisiae* npr2)	S522	
An0399/NrtB	S252, S255, S259, S260	T247, S255
AN5134/GltA	S682, S1925	
An4376/GdhA	S397	S397
AN7451/GdhB	S581, S582	
AN10709/GfaA	T90, T93, S95, T238, T312, S313	T238, T312, S313
AN4159/GlnA		S316
AN10019/OcA2	S109, T112, S205, S206, T211, S224, S225, S649	S205, S206, S210, T211, S224, S225, S323

Table S7. H_2O_2-induced phosphorylation changes in proteins involved in proteosome-mediated proteolysis

Protein	Phosphosites without H_2O_2	Phosphosites with H_2O_2
AN1966/HulE	S169, S313, T1201, S1205, T1210, S1629, S1630, S1634, S3064, T3067, T3708	S169, Y1113, T1121, T1201, S1203, S1205, T1210, T3708

AN2267 (Ubiquitin C terminal hydrolase family protein)	S337, S483, S576, S618, S621, T622, S623, S641, S677	S337, S483, S484, S576, S677
AN2072 (Putative ubiquitin specific protease; ortholog of *S. cerevisiae* Doa4p)	S160, S161, S359, T368	S160, S161, T368
AN4170/CreD	T383, S508, Y509, S510	S388, T392, S508, S510
AN2442 (Ubiquitin protein ligase (Asi3), putative)	S699, T761, S776, T777, S788	S776, S777, S788
AN6359/SconB (part of the ubiquitin ligase complex/ involved in sulfur metabolite repression)	S271, S278, S303,	S115, S271, S278, S281
AN1995 (Ubiquitin ligase)	T595, S596, S601	T595, S601
AN3923 (Ubiquitin protein ligase activity)	S1294	S1294
AN7422 (Ubiquitin carboxyl-terminal hydrolase)	S350	S350
AN6164 (Thiol-dependent ubiquitinyl hydrolase activity)	S93, T95, S1038, S1901, T1902, S1903	
AN3597/AcrB		S910, S911
AN5186 (Thiol-dependent ubiquitin specific protease activity)	S205, S786	
AN3711 (Molecular adaptor activity, thiol-dependent ubiquitin specific protease activity)	S140, S141	
AN6913 (Thiol-dependent ubiquitin specific protease activity)	S126	
AN1339/HulA	S464	
AN3587 (Ubiquitin processing protease involved in carbon catabolite repression)	S258, S260, S261, T269	
AN0883 (Ubiquitin conjugating enzyme)	S142	
AN1700 (Putative 26S proteasome regulatory subunit)	T182, S184, S940, T946, T948, T949	T182, S184, S940, T946, T949
AN5607 (Peptidase activator activity, proteasome binding activity)	T25, S26, T20, S1858, S1861, S1862, S1865, T1870	S1858, S1861, S1862, S1865
AN5872 (Proteasomal ubiquitinin-dependent protein catabolic process)	S16	
AN6547 (Proteasomal ubiquitinin-dependent protein catabolic process)		S110
AN8192/UlpB	T500, S501	S167, S496, T500, S501
AN10456/DenA		S241, S243, S253
AN2689/ UlpA	S595	

AN3449 (Thiol-dependent ubiquitin specific protease activity; ortholog of *S. cerevisiae* Otu1)		S97, S98

Table S8. H_2O_2-induced phosphorylation changes in proteins involved in gluconeogenesis and the pentose phosphate pathway

Protein	Phosphosites without H_2O_2	Phosphosites with H_2O_2
AN4462/PycA	S40, S1111	S40
AN5604/**AcuG**	S323	S323
AN2875/FbaA		S8
AN2688 (Glucose 6 phosphatase activity, sphingosine 1 phosphatase, Ortholog of *S. cerevisiae* Sgp1)	S387, S410, S418, S443	S387, S410, T430, S443
AN2867/PgmB	T106, T112, T114	S114
AN0689/FacB	T653, S655	T653
AN6499/MdhC		S326
AN1059/facC	S62, S707, S708	S707, S708
AN0688 (Putative transketolase, ortholog of *S. cerevisiae* Tkl1)	S255, S335, S674	S335, S362, S674
AN9180 (Putative transketolase, ortholog of *S. cerevisiae* Tkl2)		S290
An0240/PppA	S38, S51, S206, S209, S 227, S268, S278	S206, S227, S278, Y279
AN6037/SwoM	S3	
AN3954 (Putative phosphogluconate dehydrogenase)	S131, S166, T249	S131, T249
AN12221/AN4772 (predicted catalytic activity, deoxyribose phosphate aldolase activity, lyase activity)	S263	S262, S263
AN6711/Prs1 (Putative ribose phosphate pyrophosphokinase)	T260, S268, S255	T260, S262, S268
AN3223/PfkA (Putative 6 phosphofructokinase with a predicted role in gluconeogenesis and glycolysis)	S331, T786, S789, S790	S784, T786, S789, S790
AN0688 (Putative transketolase, ortholog of *S. cerevisiae* Tkl1)	S255, S335, S674	S335, S362, S674
AN3169/Prs3 (Putative ribose phosphate pyrophosphokinase)	S121, S128, S129, S131, S132, S164, S165, S167, S178	S164, S165, S167, T182, S185
AN1015/Putative phosphorylase with a predicted role in glycogen degradation, ortholog of *S. cerevisiae* Gph1)	S11, T37	S11, S17, T37
AN3829/Uga2		T261, T262
AN1923/Alt1		S72

AN8990 (GABA transporter, putative)	S23, S531, S532	S532

Table S9. H_2O_2-induced phosphorylation changes in proteins directly involved in ROS metabolism

Protein	Phosphosites without H_2O_2	Phosphosites with H_2O_2
An3973/TpxB		S177
AN10223/TpxA	S136	S136
AN0170/TrxA	S83	S83
PrxA/An8692	S30	
An2981/GsdA		S418
AN9339/CatB		S719
AN5918/CatC	S15	S15
AN10660/ndiA		S261
AN6046/NoxR	S308, T309, T310	S308, T309
AN3004/OXR1	S315, T317, T318, T319	
AN1100 (Hypothetical 5-oxoprolinase; similar to *S. cerevisiae* Oxp1)		T318, S322, T324
